# Traffic networks are vulnerable to disinformation attacks

**DOI:** 10.1038/s41598-021-84291-w

**Published:** 2021-03-05

**Authors:** Marcin Waniek, Gururaghav Raman, Bedoor AlShebli, Jimmy Chih-Hsien Peng, Talal Rahwan

**Affiliations:** 1 Computer Science, Science Division, New York University Abu Dhabi, Abu Dhabi, United Arab Emirates; 2 Department of Electrical and Computer Engineering, National University of Singapore, Singapore, Singapore; 3 Computational Social Science Lab, Social Science Division, New York University Abu Dhabi, Abu Dhabi, United Arab Emirates

**Keywords:** Computer science, Civil engineering

## Abstract

Disinformation continues to raise concerns due to its increasing threat to society. Nevertheless, a threat of a disinformation-based attack on critical infrastructure is often overlooked. Here, we consider urban traffic networks and focus on fake information that manipulates drivers’ decisions to create congestion at a city scale. Specifically, we consider two complementary scenarios, one where drivers are persuaded to move towards a given location, and another where they are persuaded to move away from it. We study the optimization problem faced by the adversary when choosing which streets to target to maximize disruption. We prove that finding an optimal solution is computationally intractable, implying that the adversary has no choice but to settle for suboptimal heuristics. We analyze one such heuristic, and compare the cases when targets are spread across the city of Chicago vs. concentrated in its business district. Surprisingly, the latter results in more far-reaching disruption, with its impact felt as far as 2 km from the closest target. Our findings demonstrate that vulnerabilities in critical infrastructure may arise not only from hardware and software, but also from behavioral manipulation.

## Introduction

The ubiquity of social media and the internet has made it easier than ever to spread disinformation^1–4^. Exacerbating this phenomenon are the recent advances in machine learning and the rise of social bots, allowing (dis)information to be delivered to a target audience at an unprecedented scale^1,5,6^. Disinformation has not only grown in reach but also in sophistication, with its manifestations ranging from counterfactual social media posts and manipulated news stories, to deep fake videos^7–12^. It is, therefore, unsurprising that disinformation has come to be considered a serious threat to society^10,13^.

Several disinformation campaigns have recently garnered significant attention from the public and the scientific community alike. Such campaigns have been used^2,4,7^ to shape narratives in the public debate on various issues including healthcare, vaccination, and climate change, to name just a few. Some have even argued that disinformation is being *weaponized* to manipulate the long-term decisions of a society^14,15^, e.g., during the 2016 US presidential elections and the Brexit campaign, when strategically-created propaganda, conspiracy theories, and fake social media posts were used to manipulate undecided voters^5,8,9^.

Nevertheless, the possibility that a malicious actor could use disinformation in a targeted attack to influence social behavior within a limited time has been rarely considered. If such an attack is plausible and indeed effective, it underscores an important but largely-overlooked vulnerability in complex systems whose behavior emerges from the collective decisions of the individuals therein. This is particularly alarming when the system under consideration is critical infrastructure, especially since the World Economic Forum has identified cyberattacks on critical infrastructure as one of the global risks faced in 2020^16^. In this context, demonstrating the effectiveness of a disinformation-based attack may have important policy implications in securing critical infrastructure, taking into consideration the possibility of manipulating social behavior.

Here, we focus on the influence of disinformation on urban road networks, where individual drivers constantly make decisions about their routes, timing, and destination, all of which collectively shape the city-wide traffic. The behavior of drivers has already been shown to contribute significantly towards creating bottlenecks in the traffic flow^17–19^. Thus, if an adversary is able to manipulate driving behaviors, they can potentially create massive disruptions and spill-over effects similar to those observed during the 2013 Fort Lee lane closure in New Jersey^20^.

## Related works

Despite the abundance of research on the resilience of traffic infrastructure to malicious attacks, current studies focus predominantly on vulnerabilities introduced by hardware and software systems. For instance, numerous studies^21–24^ have demonstrated how crucial control systems of modern vehicles, e.g., steering, braking, and engine control, could be hijacked, particularly in vehicles connected to external communication networks^25–29^. The consequences of such attacks include unpredictable vehicle behavior that could lead to accidents. Other studies have shown that traffic in a city could be disrupted by fooling intelligent algorithms that control traffic signals^30–39^, either by hacking into the signal control software or injecting false data through hacked vehicles. Similar attack strategies can be used to manipulate traffic navigation applications as well; see, e.g.,^40^. Automated, or self-driving vehicles have also been shown to harbor several vulnerabilities^41,42^. For instance, such vehicles can be misled by defacing road signs in subtle ways^43^. This way, without tampering directly with the vehicles themselves, an adversary could manipulate the computer vision algorithms employed by such vehicles and alter their driving patterns. Another class of security problems considered in the literature are denial-of-service attacks on ride-sharing services^44^. Note that all of the aforementioned threats arise from either the software or hardware vulnerabilities of the vehicles and traffic signaling systems, whereas our study focuses on behavioral vulnerabilities. In particular, we ask the following questions: Can an adversary disrupt traffic at a city scale without tampering with vehicle or traffic control systems, but focusing entirely on driver behavior manipulation?

## Results

### Problem overview and survey study

We consider a scenario where an adversary spreads false information with the aim of manipulating the routes taken by drivers in a city. One way to do this would be to persuade drivers to move away from a certain location, either by sending fake traffic alerts informing them of an accident or heavy congestion at that location, or by deploying fake road signs redirecting traffic away from it. An alternative way in which the adversary may disrupt traffic is by manipulating drivers to move towards a target location, e.g., by sending fake alerts advertising a limited-time, massive sale at a popular retail store. We term the former scenario a “*divergence attack*”, and the latter a “*convergence attack*”. In both cases, a proportion of the recipients may follow-through on the disinformation, i.e., reroute their trips accordingly, while the remaining recipients simply ignore it and maintain their original routes.

**Figure 1:**
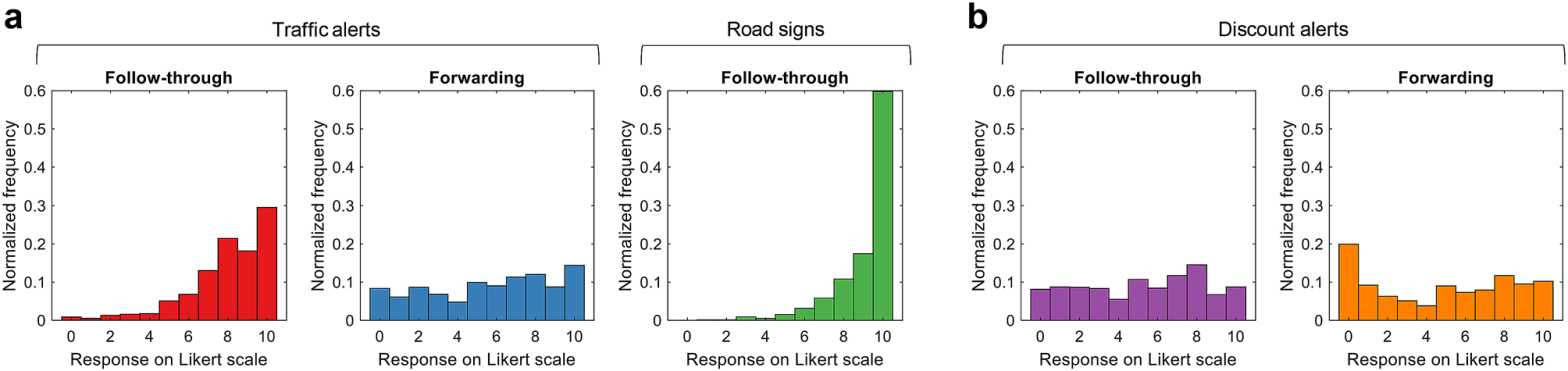
Assessing propensities to follow-through and forward disinformation. (**a**) Survey results for the divergence attack, represented as a normalized histogram summarizing the participants’ responses on a Likert scale from 0 to 10 indicating their propensity to follow-through on and forward fake traffic alerts ($n=3301$), and follow-through on fake road signs ($n=1000$). (**b**), The same as (**a**) but for convergence attacks via fake discount alerts ($n=1000$).

We begin by running surveys to assess how people would react to the disinformation involved in such attacks. Although peoples’ responses in a real-life situation may not be exactly what is reported in the survey, these responses nevertheless give us an insight into how receptive people are towards such disinformation. Starting with the divergence attack, we ran a survey on Mechanical Turk ($n=3301$), where each participant was shown an SMS notification whose sender is *‘SMSAlert’* and whose content is: *“Accident on ‘X’ Road. Please use alternative routes. Be safe!”*; see SI Appendix, Fig. S1 for an illustration of this message. The participants were then asked to specify on a Likert scale from 0 to 10 their likelihood to follow-through on the notification. Moreover, since the reach of the disinformation may increase if the recipients forward its content to others, e.g., via social media, the participants in our survey were also asked to specify their likelihood to forward the notification to their friends. Furthermore, to test peoples’ reactions when the disinformation is conveyed via a road sign, we surveyed $n=1000$ participants who were shown a picture depicting a road sign saying *“ROAD AHEAD CLOSED”*; see SI Appendix, Fig. S2. The results are summarized in Fig. 1a. Worryingly, the histograms of the follow-through responses are skewed to the left, regardless of whether the disinformation medium is a traffic alert or a road sign. In particular, the percentage of who report their propensity to follow-through to be 6 or above is 89% in the case of traffic alerts, and 97% in the case of road signs. On the other hand, when assessing people’s propensity to forward the SMS notification, we found that only 55% of participants report a propensity of 6 and above, implying that people are more likely to follow-through than to forward the notification. Overall, our surveys suggest that a divergence attack would alter the behavior of the majority of recipients. Admittedly, this significant propensity to believe the message may be attributed to the participant’s belief that the message originated from a legitimate source, although it should be emphasized that the participants in our survey were given no information regarding the identify of the sender other than the fact that its name is ‘SMSAlert’. Note that some mobile service providers do offer the option of messaging a large number of individuals, e.g., for advertising purposes, and allow the sender to specify their name, e.g., the name of the company behind the advertisement. Using such services, the adversary can indeed specify their name as ‘SMSAlert’ while messaging a large number of individuals. As for the convergence attack, we recorded the responses of $n=1000$ survey participants who were shown an SMS notification saying *“Mega sale TODAY only! 30% to 75% off all items in Target!”*; see SI Appendix, Fig. S3. The reported follow-through and forwarding propensities are summarized in Fig. 1b. Although people are less likely to alter their behavior when compared to the divergence attack, the percentages of people who report a propensity of 6 or above—50% for following-through and 47% for forwarding—are still worrisome. This is because of the nature of the convergence attack, where only a small increase in the number of vehicles may be sufficient to cause major disruptions since all of them will be heading towards the same location. Nevertheless, given that survey participants in general may over-report their propensity to take certain actions, we do not restrict our analysis to the survey responses, but rather consider a wide range of possible follow-through rates.

### Theoretical analysis

Naturally, the impact of such behavioral manipulation attacks not only depends on the follow-through rate, but also on the target’s location, e.g., targeting an already-congested area is likely to cause more disruption than targeting a remote area. Essentially, the adversary must solve an optimization problem which involves choosing the target that maximizes the traffic disruption. Next, we study the optimization problem corresponding to the divergence attack. We analyze the computational complexity of this problem to understand the theoretical limits of the capability of such an adversary. In our analysis, we consider a budget specifying the maximum number of targets, to account for the possibility that the adversary may attack multiple targets at the same time, which may result in a compound impact on traffic. Formally, we represent the road network as a directed graph, $G=(V,E)$, where *V* is the set of nodes, each representing an intersection or a road end, and *E* is the set of directed edges, each representing a single direction of traffic movement, i.e., a one-way street is represented by a single edge while a two-way street is represented by two separate edges. We then define $R=\{r_1,\ldots ,r_{|R|}\}$ as the set of vehicle rides, each specifying the start node, the end node, and the time of day when the ride starts, but not specifying the route itself. Now, let *b* be the “budget”, i.e., the maximum number of targets that the adversary can afford to attack. Finally, given a traffic model, *M*, let *f* be the objective function, which encapsulates the quality of traffic into a single number. Then, the optimization problem faced by the adversary involves removing at most *b* streets from the graph, *G*, in order to minimize the traffic quality, *f*, given the rides in *R* whose routes are determined based on the traffic model *M*; see SI Appendix, Definition S1. We call this the *problem of Maximizing Disruption*. Note that this formulation is only for the divergence attack, and we do not consider an analogous formulation for the convergence attack. This is because, unlike the former attack, the latter does not introduce any changes to the graph *G*.

In our complexity analysis of the problem of Maximizing Disruption, we focus on one particular objective function, $f^*$, that reflects the average time taken to complete each ride in *R*; see Definition 1 in “Methods”. We also focus on a cellular-automata traffic model, $M^*$, which is used to simulate the rides in the city; see “Methods”. Our theoretical analysis shows that given the objective function $f^*$ and the model of traffic $M^*$, the problem of Maximizing Disruption is at least as hard as any NP (non-deterministic polynomial time) problem, implying that no known algorithm can solve it in polynomial time; see Theorem 1, the proof of which is detailed in SI Appendix, Note 5.

#### Theorem 1

*The problem of Maximizing Disruption is NP-complete given the objective function* $f^*$ *and traffic model* $M^*$.

To determine whether this computational intractability arises from the complexity of the traffic model $M^*$, we consider a simpler alternative, $M^\varnothing$, which completely disregards the interdependencies between the rides; see Definition 2 in “Methods”. Surprisingly, even for this simple model, the computational complexity persists; see Theorem 2.

#### Theorem 2

*The problem of Maximizing Disruption is NP-complete given the objective function* $f^*$ *and traffic model* $M^\varnothing$.

Next, we analyze the computational complexity of a slightly different problem, which involves identifying the minimum number of targets needed to achieve a predetermined level of disruption in a divergence attack; we call this the *problem of Minimizing Targets*, see SI Appendix, Definition S4.

#### Theorem 3

*The problem of Minimizing Targets is APX-hard given the objective function* $f^*$ *and either traffic model* $M^*$ *or* $M^\varnothing$. *In particular, the problem does not admit a polynomial-time approximation scheme (PTAS) unless P = NP*.

Our analysis shows that this problem is APX-hard given both our model of traffic $M^*$ and the simple model $M^\varnothing$. Intuitively, it is computationally intractable to approximate, let alone find, the optimal solution. One of the consequences of this finding is that the problem does not admit a polynomial-time approximation scheme (PTAS). In the computational complexity literature, a PTAS is an algorithm that takes a parameter $\epsilon >1$ and, in polynomial time, finds a solution that is within a factor $\epsilon$ of an optimal solution. Our computational complexity results are summarized in Table 1.

**Table 1:** Summary of our computational complexity results.

Model of traffic	Maximizing Disruption	Minimizing Targets
$M^*$	NP-complete	APX-hard
$M^\varnothing$	NP-complete	APX-hard

Given our complexity analysis for the divergence attack, we can safely assume that the adversary will not be able to identify an optimal set of targets in the network, and has no choice but to settle for a suboptimal solution. With this in mind, we analyze a *greedy* heuristic where the adversary starts by computing the set $\Pi$ of all shortest paths between any two nodes in the graph *G*. The heuristic then proceeds in iterations, each targeting an edge that appears in the largest number of paths, taking into consideration only the paths in $\Pi$ that were not already affected by previous iterations. The fact that this greedy heuristic selects one target at a time, rather than selecting them all at once, allows the solution to be computed much more efficiently, although this may come at the expense of solution quality. Note also that in order to perform this attack the adversary only needs to know the structure of the city’s road network, as the algorithm does not require any information about the traffic on a given day. An alternative approach would be to perform the attack that maximizes the number of rides affected by the edge removal. Nevertheless, performing such an attack would require information about the paths of all the rides, and, consequently, a very strong assumption about the knowledge of the adversary.

**Figure 2:**
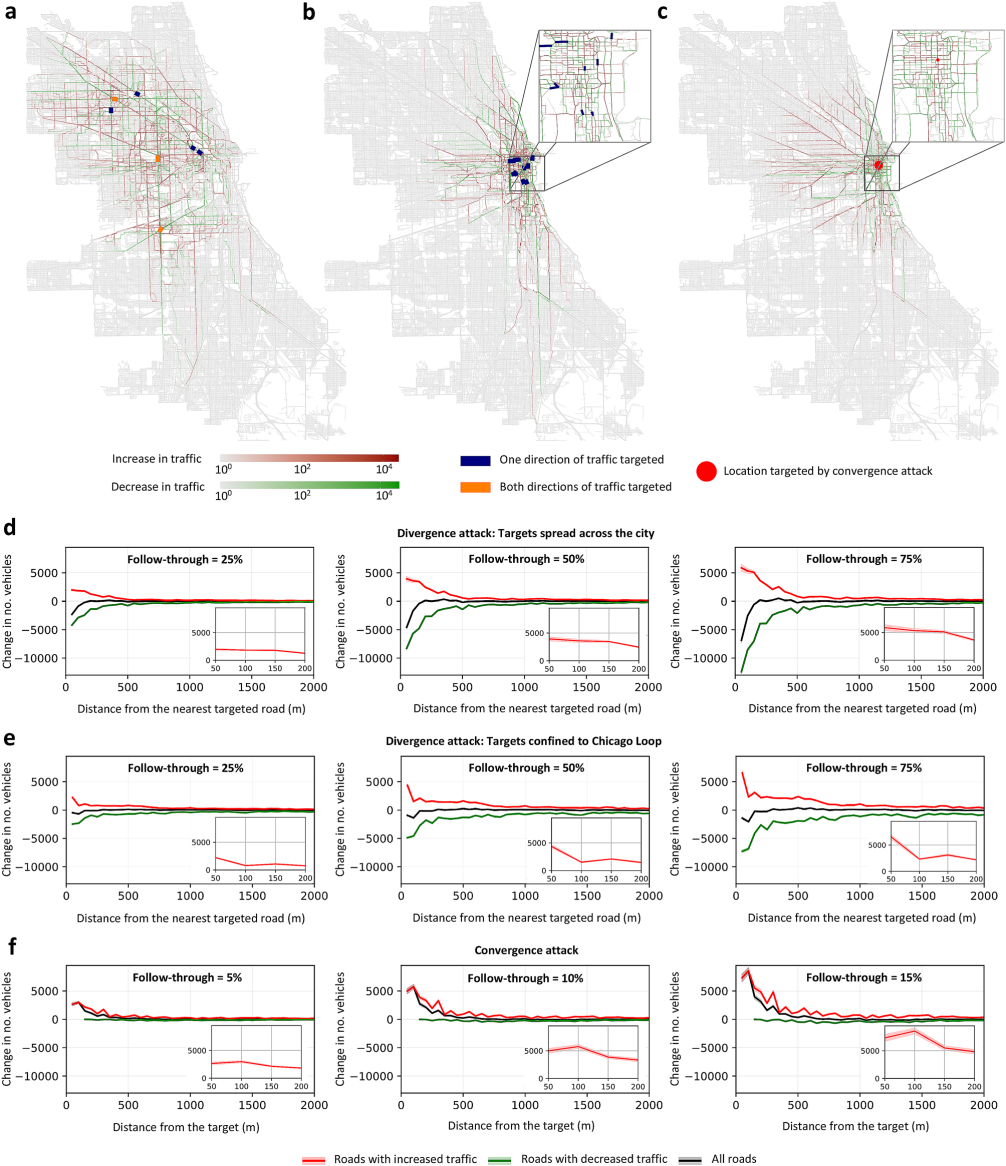
Impact on streets across the city. (**a**) Given a divergence attack with 10 targets, thick lines indicate the streets targeted by our greedy heuristic. Dark blue indicates streets where only a single direction of traffic is targeted, whereas orange indicates streets that contain two targets that cover both directions of traffic. For all remaining streets, the color indicates the change in the number of vehicles passing through when the follow-though rate is 50%, with green indicating fewer vehicles, red more vehicles, and grey no change. (**b**) The same as (**a**) but the greedy heuristic is now confined to a particular neighborhood in downtown Chicago, namely, the Loop. (**c**) The same as (**a**) but instead of a divergence attack, we now have a convergence attack on the store called “Target” located in downtown Chicago. (**d**) The change in traffic per day as a function of the distance (in meters) from the nearest target, given the targets shown in (**a**) and different disinformation follow-through rates. The black plot corresponds to all roads, whereas the red and green plots correspond to only those roads where the traffic increased and decreased, respectively. The inset figures present a zoomed-in view of the red plots in the near-vicinity of the targets. Results are averaged over 100 simulations, with the shaded areas representing the 95% confidence intervals. (**e**) The same as (**d**) but for the target locations shown in (**b**). (**f**) The same as (**d**) but for the convergence attack on the location shown in (**c**). The maps were generated using iText PDF library version 5.2.0^45^.

### Attack impact on the road network

To evaluate this heuristic, we consider a divergence attack on 10 locations in the city of Chicago. We simulate one day of traffic in the city, with and without the attack, and report the observed differences in traffic throughout the day, assuming that the attack happens at the beginning of the day and lasts for 24 h; see “Methods” for more details. Figure 2a shows the targets chosen by the greedy heuristic (for a larger version, see SI Appendix, Note 6). As can be seen, these targets are spread across the city, and not grouped in any particular neighborhood. Moreover, recall that our setting considers each direction of traffic in a street to be a separate potential target; we see from the figure that the heuristic chose to target both directions of traffic in certain locations. Given these targets, and assuming that 50% of the drivers follow-through on the disinformation by rerouting their path to avoid the targets, Fig. 2a also depicts the change in the traffic intensity as a result of the attack, i.e., the change in the number of vehicles that traverse through each street. It can be seen that the attack diverts traffic away from the targets into neighboring streets, thereby resulting in increased traffic in certain streets (shown in red), and decreased traffic in others (shown in green). Interestingly, the impact of the attack is not confined to small neighborhoods around the targets, but rather propagates across the city.

We then investigate the possibility that instead of targeting the entire city, the adversary might concentrate the attack on one critical neighborhood, e.g., its business district, in order to cause the maximum disruption in this area. To simulate this scenario, we constrained our greedy heuristic to only target locations in the Chicago Loop, which is the central business district of the city. Fig. 2b highlights the locations chosen by the modified heuristic along with the resultant impact on traffic. As can be seen, when compared to Fig. 2a, the impact of this concentrated attack is largely centered around the targeted neighborhood. While one may expect that most streets in this neighborhood will experience an increase in traffic, the zoomed-in part of Fig. 2b shows that this is not the case, as evident by the many green streets therein. This demonstrates that the heuristic can easily be modified to control the region, but not the streets, where the traffic disruption will be concentrated. Having analyzed a divergence attack on the city of Chicago, we now study the impact of a convergence attack. To this end, we consider the “Target” retail store located in downtown Chicago, and focus on vehicles whose route involves passing through a location that falls within a 1 km radius from this store. We vary the percentage of such vehicles that reroute their journeys to take advantage of the purported discount; this percentage is referred to as the follow-through rate. Furthermore, for any such follow-through rate, we add 1000 new rides originating from randomly chosen locations in the city and terminating at the target; see “Methods” for more details. The results are presented in Fig. 2c, given a follow-through rate of 10%, which is about 20,000 vehicles. As can be seen, the resulting disruption to traffic is significant around the target, and decreases as we move away from it. Larger versions of Fig. 2b,c are presented in SI Appendix, Note 6.

To further understand how the impact propagates from the targeted locations, be it for the divergence or the convergence attack, we plot the change in the number of vehicles in different streets as a function of the distance from the nearest target, while varying the disinformation follow-through rate. Since our traffic simulation is non-deterministic, we took an average over 100 simulations; the results are depicted in Fig. 2d,e for the divergence attack, and Fig. 2f for the convergence attack. The overall trend (depicted in black) is further disaggregated into the streets that experience lighter traffic (green) and those that experience heavier traffic (red). The insets zoom in on the latter category of streets within 200 m from a target, to facilitate comparison across the different settings. Starting with the divergence attacks (Fig. 2d,e), we observe that the closer a street is to a target, the greater is the impact on traffic, regardless of whether it is an increase (red plot) or a decrease (green plot) in the number of vehicles. The figures also show that higher follow-through rates result in greater impact on traffic. Comparing the insets of Fig. 2d,e reveals that when the targets are concentrated in one neighborhood the congestion is higher within 50 m from the targets, e.g., given a 75% follow-through rate, the number of vehicles throughout the day increases by about 6500 when the targets are concentrated, as opposed to approximately 5900 when they are not. However, as the distance from the nearest target increases, the impact on traffic fades away at a greater pace when targets are concentrated, e.g., at a distance of 100 m from the targets and given a 75% follow-through rate, the number of vehicles throughout the day increases by about 2300 when the targets are concentrated, as opposed to approximately 5300 when they are not. Commenting on the reach of the disruption caused by the divergence attack, we find that as the follow-through rate increases, the disruption extends farther away from the targets. We also find that the reach is greater when the targets are concentrated, with a considerable impact being felt as far as 2 km away from the closest target in the case of 75% follow-through rate. This could be due to the synergy between the different targets when they are within close proximity to one another. Having discussed the divergence attacks, we now analyze the propagation of the impact due to the convergence attack. Referring to Fig. 2f, there are two observations. First, as expected, increasing the follow-through rate results in a higher disruption. Second, although some streets experience an increase while others experience a decrease in traffic, the increase seems much more significant while the decrease seems negligible; this trend is not evident in Fig. 2c since the colors therein correspond to a logarithmic scale, making it difficult to visually infer the magnitude of the change in traffic.

### Attack impact on rides in the city

So far, we have studied the impact of the disinformation attack on the streets across the city, showing that some streets experience increased traffic, while others experience lighter traffic. However, we still do not know the distribution of the delays (i.e., the additional ride time) experienced by the individual drivers. We address this point taking into consideration two complementary scenarios: one where the goal of the adversary is to disrupt traffic *globally*, and another where the goal is to disrupt traffic *locally*. For the former, we will consider the divergence attack where the greedy heuristic is not constrained to any particular neighborhood. As for the latter, we will consider the convergence attack, which inherently focuses on a particular neighborhood of interest.

**Figure 3:**
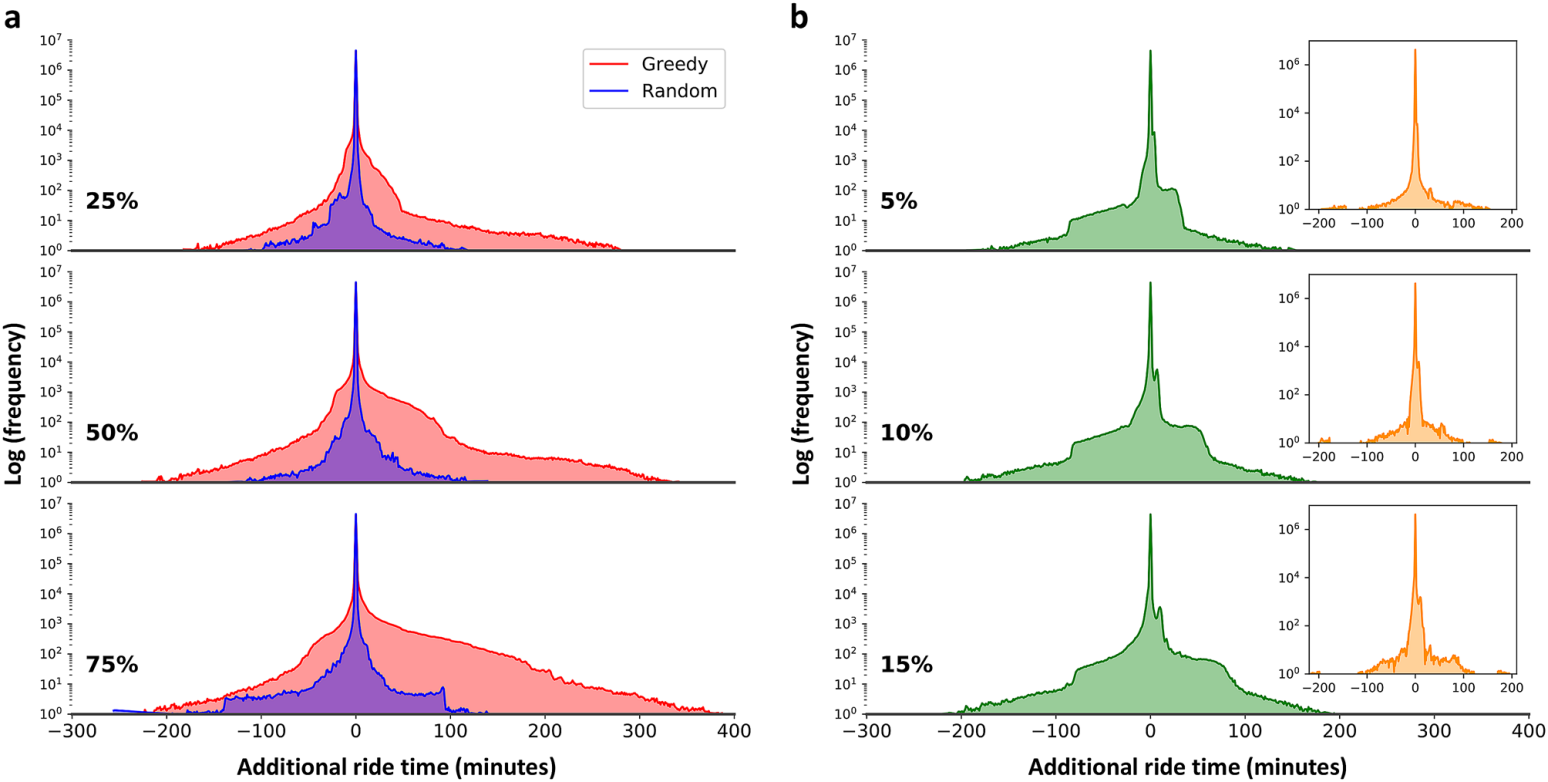
Impact on rides across the city. (**a**) Given varying disinformation follow-through rates (indicated in bold), the distribution of the additional ride time experienced by drivers after a divergence attack on 10 targets across the city, chosen either by our greedy heuristic or at random. (**b**) The same as (**a**) but for convergence attacks. The inset depicts the same distribution but instead of considering all rides, it considers only those that do not pass within 1 km of the target location. All results are shown as an average over 100 simulations.

Starting with the divergence attack, we plot this distribution considering 10 targets chosen by our greedy heuristic while varying the follow-through rate; see Fig. 3a. As a baseline, we also depict the impact caused by a *random* attack, i.e., a scenario where the adversary chooses the targets randomly. There are three observations that can be gleaned from these distributions. First, regardless of the type of the attack and the follow-through rate, the ride time increases for some drivers and decreases for others. This might be surprising at first glance, as one would expect the removal of edges from a road network to only have a negative impact on travel time. This, however, is not always true, due to what is known as Braess’ paradox^46^, a phenomenon where the removal of some streets from a network has the counter-intuitive effect of reducing the ride times for some drivers; there is theoretical^47^ and empirical^48^ evidence suggesting that this phenomenon is not uncommon. Second, increasing the follow-through rate increases the variance of the time-delay distributions, with more vehicles experiencing longer delays. For instance, when the follow-through rate is 25%, around 1100 rides suffer a delay of 60 min or more. However, when the follow-through rate is increased to 75%, this number increases to about 29,300. Third, regardless of the follow-through rate, the distribution tends to be skewed to the left in the case of the greedy heuristic, and to the right in the case of the random attack. For example, given a follow-through rate of 50%, the greedy heuristic results in 9.28% of the drivers experiencing a delay, and 7.33% a speed up of their journeys. However, for the random attack, 5.49% experience a delay while 5.57% experience a speed up. This underscores the importance of the strategy that the adversary uses to select targets of a divergence attack.

Next, we analyze the distribution of the delay experienced by the rides given a convergence attack and different follow-through rates; see Fig. 3b. Here, as with the divergence attack, we also find that the ride time becomes longer for some, and shorter for others. This is because when drivers reroute their paths to move towards the target, their original routes become less congested. One possibility is that the rides that come close to the target become slower, and those that do not become faster. Another possibility is that all the affected rides (becoming either slower or faster) are those that come close to the target, implying that the remaining rides are not affected by the attack. To determine which of these possibilities is true, we plot the distribution of the rides that *do not* pass within a 1 km radius from the target; see the insets in Fig. 3b. As can be seen, the distribution is not skewed, regardless of the follow-through rate, and the vast majority of the rides experience almost no delay (note the logarithmic scale in the Y-axis).

## Discussion

Let us first summarize the findings of our study. We have demonstrated how disinformation can be weaponized by an adversary to influence drivers’ decisions, thereby causing major traffic disruptions at a city scale. Specifically, we considered a scenario where an adversary spreads false information about a purported accident or heavy congestion in specific locations in the city, with the aim of redirecting traffic and creating congestion in nearby areas. In the survey study we evaluated the propensity of drivers to follow-through with the fake notifications, finding that a worryingly large percentage of participants express willingness to modify their driving behavior. In our theoretical analysis we showed that the problem of identifying the targets that would cause the maximum damage is not only impossible to solve optimally, but also difficult to even approximate. Further, with the use of simulations, we analyzed two separate cases where the targeted locations were either (1) spread across the city, or (2) confined to a particular neighborhood, and showed that the latter could be used to focus the disruption in critical neighborhoods of the city such as its business district. A number of vulnerabilities in traffic networks have already been considered in the literature, ranging from hacking traffic signals^30,32^, to defacing traffic signs to fool the image recognition algorithms used by self-driving cars^43^. Our study contributes to this literature by highlighting a new vulnerability—the possibility of using disinformation to manipulate drivers’ behavior at scale.

While our study is the first to consider behavioral manipulation attacks on traffic networks, it nonetheless has a number of limitations. In particular, we ran surveys and simulations to understand peoples’ propensities to follow-through and forward fake traffic alerts, and assessed the impact that such disinformation can have on traffic. Admittedly, this approach is not ideal, since people’s responses in the survey may not accurately reflect their behavior in reality. Another limitation is that we focus on shortest paths when computing the routes of the rides, which is not very realistic since people tend to choose routes that minimize travel time as opposed to travel distance. A more accurate alternative would be to compute fastest paths and update them periodically to take into consideration the constantly changing state of traffic. In such a setting, a necessary assumption would be that the attack is performed via a navigation application used by the drivers who follow-through. In graph theoretic terms, instead of assigning to every edge a weight that represents the length of the corresponding street, the weight can represent the expected time of traversing that street. However, this makes the model significantly more complex since the weights may change in every one of the 86,400 time steps in our simulation, and with each change, we may need to recalculate the affected routes. This is particularly challenging since there are over $5\times 10^6$ rides in our simulation. Even if we recalculate the routes only once every minute (i.e., every 60 time steps), then given the average ride duration in our simulation (which is 22.59 min, or 1355 time steps), we would still need to perform over $10^8$ recalculations to simulate 24 h of traffic in a network consisting of nearly 80,000 nodes and 235,000 edges. This makes running even a single simulation computationally demanding, and the situation becomes worse when the analysis requires hundreds of simulations, as is the case in Figs. 2 and 3. By focusing on distance, we significantly reduce the computational overhead, while making sure that the generated paths: (1) have an average duration that closely matches the value observed in real life; (2) reproduce the observed traffic frequency in different locations across the city; and (3) reproduce the temporal distribution of the traffic intensity throughout the day; see “Methods” for more details. One may consider a completely different approach; instead of a simulation-based study, one may perform a field experiment where actual drivers are sent fake notifications, and then observe and analyze the resultant impact on traffic flows. However, the main challenge in performing such an experiment would be the ethical and safety considerations involved in manipulating such a large number of unwitting drivers. What is more, it is likely that running such an experiment just once would not yield a sufficient amount of data, since the results in the article are presented as an average over hundreds of simulations.

Our study has a number of implications. First, as suggested by the high propensities reported in our survey, people seem likely to follow-through on fake traffic notifications. Perhaps the reason behind such high propensities is the seemingly harmless nature of these alerts. After all, the alerts do not attempt to extract any information from the recipient, e.g., by asking them to click on suspicious external links, unlike the case with phishing attacks and spam. Second, given the disruptive impact of the attack, it is imperative to detect and effectively counter such disinformation. While it may be difficult for individuals not physically present near a purported traffic incident to judge its veracity, one plausible solution would be to crowdsource this verification process to those who happen to be close to it. Crowdsourced fact-checking has already been shown to be very effective in identifying more vs. less reliable news sources^4^. However, out of all commonly-used navigation applications, only one offers such a functionality, namely Waze^49^, which serves about 11% of all users in the US^50^. One clear policy implication of our study is to extend this functionality to all other navigation applications.

There are a number of potential directions to expand our work. First, a possible venue of future research involves traffic simulations where some or all of the rides utilize navigation systems that dynamically compute shortest time routes and can react to attacks taking place throughout the simulation. Second, while we assumed that the adversary knows the structure of the road network, but not the current distribution of rides, investigation of adversaries with different levels of knowledge about the system is also possible. Finally, an important direction is the analysis of other scenarios where security vulnerabilities arise from neither hardware, nor software, but rather from the collective behavior of the system users.

In conclusion, we have shown that in the age of disinformation, an adversary no longer needs to tamper with traffic control systems, but can instead focus entirely on manipulating the drivers themselves to disrupt traffic in a city. More broadly, our study provides a new perspective on the security of critical infrastructure, demonstrating that vulnerabilities can emerge not only from the hardware and software, but also from the behavior of the individuals interacting with the system.

## Methods

### Definition 1

($f^*$) Given a graph *G*, a set of rides *R*, and a traffic model *M*, the objective function $f^*$ is computed as:

$$\begin{aligned} f^*(G,R,M) = \frac{1}{|R|} \sum _{r_i \in R} \frac{1}{\mathcal {T}(r_i,G,M)} \end{aligned}$$

where $\mathcal {T}(r_i,G,M)$ is the time taken to complete the ride $r_i \in R$ in the graph *G* according to the model *M*.

### Definition 2

($M^\varnothing$) Given a graph *G*, and a set of rides *R* where every ride $r_i \in R$ travels from a starting node $w^{start}_i \in V$ to a destination node $w^{end}_i \in V$, the time of travel according to the simple traffic model $M^\varnothing$ is:

$$\begin{aligned} \mathcal {T}(r_i,G,M^\varnothing ) = d_G(w^{start}_i,w^{end}_i) \end{aligned}$$

where $d_G(w^{start}_i,w^{end}_i)$ is the number of edges on a shortest path between the two nodes, unless there exists no path between them, in which case $d_G(w^{start}_i,w^{end}_i)=\infty$.

### Road network generation

We obtained the road network data of Chicago from OpenStreetMap (OSM)^51^. However, we could not directly utilize this data for our simulations since: (1) some parts of the network were either disconnected or weakly connected due to the fact that OSM is crowdsourced and some streets were left unreported; (2) the data did not contain information about the number of lanes in each edge of the network, which is required in our traffic model $M^*$; and (3) a section of a road may be represented by multiple edges in OSM instead of a single edge, making the corresponding graph *G* needlessly large, thereby increasing the processing time in our simulations. Based on these observations, we developed an algorithm that takes the OSM data as input and generates a road network that addresses all of the aforementioned issues; see SI Appendix, Note 2 for more details.

### Ride generation

We generated the set of rides *R* (each ride being defined by its start point, end point, and time of the day when it begins) in Chicago by combining: (1) publicly reported data about the number of vehicles passing by different locations in Chicago in different days of the year^52^, with (2) the daily traffic intensity distribution of Chicago provided by the Texas A&M Transportation Institute^53^. We considered the number of vehicles corresponding to the month of October as this month had the greatest number of data points. As for the daily traffic intensity, we use an average taken over all weekdays. The detailed algorithm for generating the rides is presented in SI Appendix, Note 2.

### Our model of traffic M*

Our model of traffic, $M^*$, is a modified version of the Nagel–Schreckenberg model^54^. We had to modify this model since it was only designed to model traffic in a single street, whereas our requirements call for modeling the traffic flows in a directed network of streets. We note that ours is not the first work to extend the Nagel–Schreckenberg model to a generic network. A similar extension was proposed by Gora^55^ whose study focused on the role of traffic lights in managing the traffic flows. However, their traffic model was presented in rather vague terms, and therefore could not be used in our study.

The model $M^*$ is a cellular-automata model, and takes as input a directed road network $G=(V,E)$ and the set of rides $R=\{r_1,\ldots ,r_{|R|}\}$. Each ride $r_i$ is of the form $(w^{start}_i,w^{end}_i,\theta _i)$, where $w^{start}_i \in V$ is the start node, $w^{end}_i \in V$ is the end node, and $\theta _i$ is the time of day when the ride starts. Each edge $e\in E$ in the network has a specified length $d_e$ and number of lanes $l_e$. Every lane of the edge *e* is further divided into a number of cells; this number is denoted by $c_e$ and is computed as: $c_e=\lceil \frac{d_e}{d_{ vehicle }} \rceil$, where $d_{vehicle}$ is the average length of the space occupied by each vehicle on the road, including the separation between two consecutive vehicles on the same lane. The model proceeds in discrete time steps. The time step corresponding to any given start time, $\theta _i$, is denoted by $\tau (\theta _i)$. In other words, $\tau$ maps any given clock time to a particular time step in the model. Each cell can be occupied by at most one vehicle in any given time step. Similar to the Nagel–Schreckenberg model, the speed of each ride, $v_i$, in our model is expressed as the number of cells it can traverse in a single time step. The maximum speed of all the rides is denoted by $v_{max}$. At the beginning of each time step, rides that start at the corresponding time of the day are introduced. For each such new ride, the shortest path in the network from its start node to the end node is calculated, and the state of the ride is set as “Waiting at the first edge of the shortest path”, i.e., waiting to be inserted into the first cell of one of the lanes in that edge, and its speed is set to zero. Subsequently, the model iterates over all rides that are waiting in the current time step (either because they just started or they reached an intersection in a previous time step), and if the edge that the ride is waiting at, *e*, contains a lane with an empty first cell, the ride is assigned to the first cell of this lane. In case there are multiple lanes with an empty first cell, one of them is chosen uniformly at random. The state of such a ride is then set to “Traversing the edge *e*”. Next, the model iterates over all the rides traversing an edge in the network, and performs the following four steps as defined by the Nagel–Schreckenberg model for each. First, the speed of the ride is increased by 1, up to the maximal speed limit $v_{max}$. Second, we determine if the ride can move forward on its current lane while maintaining its current speed, e.g., if its speed is 3, then, we check if there are 3 empty cells in front of it. If the number of empty cells is smaller than the speed, the ride is changed to another lane that does offer the necessary empty cells. If no such lane exists, then the ride is changed to the lane that has the maximum number of empty cells, and the speed is reduced to match the available number of empty cells in that lane. Third, with a fixed probability $p_{slow}$, the speed of the ride is decreased by 1 to simulate random events. Finally, the ride is moved forward by the number of cells equal to its speed. If the ride reaches the end of the edge that it is currently on, it is removed from the edge, and its status is set to either “Waiting at the next edge of the shortest path” (if the ride has more edges to traverse) or to “Finished” (if the ride reached its final destination). Note that reaching an intersection in the real world corresponds to reaching the end of the edge in our model. The process continues until a number of time steps $t_{max}$ are completed and all the rides in *R* reach their destination. For more details and the pseudocode for the model, see SI Appendix, Note 3.

In our study, we assume that each time step in the model corresponds to 1 s, i.e., $\tau (\theta _i) = \theta _i$. We further set $d_{vehicle} = 7.5$ metres and $v_{max}=5$, which are the standard values used in the Nagel–Schreckenberg model^56^. These parameters yield a maximum speed of 84 mph, since a vehicle can in 1 s traverse up to 5 cells, each of which is 7.5 m long. The probability that a ride slows down is taken as $p_{slow}=\frac{1}{100}$. Finally, we set $t_{max}=$ 86,400, which is the number of seconds in 24 h. To assess whether our model generates realistic rides, we ran 100 simulations of traffic in the Chicago network, with each simulation considering a new set of rides generated according to the procedure described above. We found that the average time of travel in the network is 22.59 min. In comparison, the American Driving Survey^57^ reported that a typical American driver in 2016 and 2017 spent 51 min driving each day, making 2.2 trips, resulting in an average ride time of 23.18 min. This close correspondence between the real data and simulation outcomes demonstrates that our model is indeed realistic.

### Traffic simulations under attack

Starting with the divergence attack, we determine the set of edges that are targeted by the adversary. This depends on the budget *b* of the attack as well as on whether the adversary uses the greedy heuristic or adopts the random approach. The targets are chosen from a set $Q\subseteq E$. If the adversary is choosing targets from across the entire city, then $Q=E$; otherwise, if the adversary is targeting a particular neighborhood (e.g., Chicago Loop in our scenario), then *Q* consists of all edges in that neighborhood. The set of chosen targets is then denoted by $Q^*\subseteq Q$. Next, the number of drivers who follow-through, i.e., those who modify their rides to avoid the targets, is determined based on the disinformation follow-through rate. The specific rides that follow-through are then chosen randomly. The route of every such ride is then computed as a shortest path in the graph $(V,E\setminus Q^*)$, thereby ensuring that the ride avoids all edges in $Q^*$, i.e., avoids all streets targeted by the adversary. As for the remaining rides, their route is computed as a shortest path in (*V*, *E*), without being constrained by the targets. We perform 100 simulations for each attack strategy (either greedy or random) and follow-through rate, using the same set of rides generated for the baseline simulation corresponding to the scenario where no attack is carried out.

Having explained how we simulate traffic under the divergence attack, we now move on to the convergence attack. Here, a single node $w^* \in V$ is chosen as the target. Next, the rides that follow-through are chosen randomly out of those that pass within a 1 km radius of $w^*$. Each such ride is then modified so that it follows a shortest path from the original starting node to $w^*$, then stops at $w^*$ for a random number of minutes ranging from 30 to 90, before following a shortest path from $w^*$ to its original destination. In addition, we add 1000 extra rides to the set of rides in our simulation, i.e., *R*. For each such ride, the starting node $w^{start}_i$ is selected according to the distance distribution $\mathcal {N}$, and the starting time is chosen according to the traffic intensity distribution $\Theta$; these two distributions are the same as those used in our ride generation algorithm, see SI Appendix, Note 2. Each newly-added ride follows a shortest path from $w^{start}_i$ to $w^*$, stops at $w^*$ for 30–90 min, and then follows a shortest path from $w^*$ back to $w^{start}_i$.

In our analysis we compare the traffic in a network under attack, with traffic in a network without the attack. Hence, we assume that the attack takes place at the beginning of the simulation and lasts until its end. We made this assumption to keep both settings easier to compare and make simulations more computationally tractable. Nevertheless, an alternative scenario could involve performing the attack during the simulation and rerouting some of the rides that start before the attack and end after the attack.

On a side note, when illustrating the network in Fig. 2a–c, to improve visibility we omitted the section corresponding to O’Hare airport, which is at the periphery of the network; see SI Appendix, Fig. S4 for the full network. Note that our simulations were run on the entire network, and the section was only omitted in the figures without loss of information since the attacks caused no traffic changes in this area.

### Ethics statement

The research was approved by the Institutional Review Boards of the New York University Abu Dhabi and the National University of Singapore. All research was performed in accordance with relevant guidelines and regulations. Informed consent was obtained from all participants.

## Supplementary Information

Supplementary Informtaion 1.

## Data Availability

The data that support the findings of this study are available at^58^.

## Code availability

The code for generating the results will be made available upon publication.
