# Supplementary material for: Traffic networks are vulnerable to disinformation attacks
Source: Sci Rep. 2021 Mar 5;11:5329. doi: 10.1038/s41598-021-84291-w (PMC7935872; doi:10.1038/s41598-021-84291-w)
Supplement: Supplementary file 1 — Supplementary Informtaion 1. [file 41598_2021_84291_MOESM1_ESM.pdf]

Supplementary Materials for

Traffic networks are vulnerable to disinformation attacks

Marcin Waniek, Gururaghav Raman, Bedoor AlShebli,
Jimmy Chih-Hsien Peng*, and Talal Rahwan*

* Corresponding authors. E-mail: jpeng@nus.edu.sg; talal.rahwan@nyu.edu

This document is structured as follows:

- **Section S1** (*page 2*) presents the survey questionnaires;
- **Section S2** (*page 10*) describes how the road network and vehicle rides in Chicago were generated;
- **Section S3** (*page 13*) describes our model of traffic M^*;
- **Section S4** (*page 15*) presents the definitions used in our theoretical analyses;
- **Section S5** (*page 16*) presents the results of our theoretical analyses;
- **Section S6** (*page 21*) presents larger versions of Figs. 2(a), 2(b), and 2(c) from the main manuscript.

S1 Note 1: Survey questionnaires

S1.1 Survey-1

We ran a survey to understand how participants react to fake notifications regarding traffic alerts. We recruited 3,301 participants through Amazon Mechanical Turk, who were then directed to a survey on the Qualtrics platform. The participants were residents of one of the 35 most traffic congested cities in the United States. The survey consisted of five steps, an overview of which is provided below. Detailed explanations are provided in Sections S1.1.1 through S1.1.5.

Step 1: A consent form is displayed (see Section S1.1.1), after which the demographic questions are asked (see Section S1.1.2);

Step 2: The following sentence is displayed:

– "*Suppose you receive the following message from SMSAlert*";

Step 3: The message containing the fake notification is displayed (see Section S1.1.3);

Step 4: The participant is asked questions regarding their follow-through and forwarding propensities (see Section S1.1.4);

Step 5: The participant is awarded a financial compensation of $0.5, and thanked for their participation (Section S1.1.5).

S1.1.1 Consent form

The consent form consists of the following text:

> *Welcome to this study investigating how humans behave upon receiving text notifications. You are eligible to participate in the study at this time if you are:*
>
> 1. *18 years of age or older;*
> 2. *live in one of the following 35 cities: Albuquerque (NM), Austin (TX), Baltimore (MD), Boston (MA), Charlotte (NC), Chicago (IL), Columbus (OH), Dallas (TX), Denver (CO), Detroit (MI), El Paso (TX), Fort Worth (TX), Fresno (CA), Houston (TX), Indianapolis (IN), Jacksonville (FL), Las Vegas (NV), Los Angeles (CA), Louisville (KY), Memphis (TN), Milwaukee (WI), Nashville (TN), New York City (NY), Oklahoma City (OK), Philadelphia (PA), Phoenix (AZ), Portland (OR), Sacramento (CA), San Antonio (TX), San Diego (CA), San Francisco (CA), San Jose (CA), Seattle (WA), Tucson (AZ), or Washington (DC).*
>
> *The questionnaire asks about your background and your reaction upon receiving text notifications. This survey is anonymous, i.e., it does not contain individually identifiable data from you. Your participation is voluntary, and you may close the survey at any point.*
>
> *The questionnaire is expected to last on average 5 minutes. An amount of $0.5 will be paid upon successful completion of the survey.*
>
> *This research is conducted by Jimmy Chih-Hsien Peng and Gururaghav Raman at the National University of Singapore, as well as Talal Rahwan, Bedoor AlShebli, and Marcin Waniek at New York University Abu Dhabi, and has been approved by the respective Institutional Review Boards. For questions about the rights of research participants, you may contact the University Committee on Activities Involving Human Subjects, New York University Abu Dhabi, irbnyuad@nyu.edu and the National University of Singapore, irb@nus.edu.sg.*
>
> *If you have any questions, suggestions or concerns, please feel free to reach out to us at nyuad.textnotification@nyu.edu – an email address that only researchers associated with this project have access to.*
>
> *Please do not complete the survey more than once. Upon finishing the survey you will receive a completion code. The payment of $0.5 will be made once you've entered that code in the space provided.* ***Please do not close the browser with your MTurk account****. If you read this consent form, and would like to participate in this study, press the button below!*

S1.1.2 Demographic questions

The demographic questions that were asked to each participant are detailed below:

1. *What is the sex listed on your birth certificate?*
   (a) *Male*
   (b) *Female*
2. *What is your ethnicity?*
   (a) *Hispanic*
   (b) *Non-Hispanic*
3. *What is your race?*
   (a) *White*
   (b) *Black or African American*
   (c) *Asian*
   (d) *Native American*
   (e) *Middle Eastern or North African*
   (f) *Mixed*
   (g) *Other*
4. *What is your age?*
   [empty field to be filled by a number]
5. *What is your highest completed level of education?*
   (a) *Less than high school*
   (b) *High school graduate or equivalent (e.g., GED)*
   (c) *Some college*
   (d) *2 year degree (i.e. Associate's degree)*
   (e) *4 year degree (i.e. Bachelor's degree)*
   (f) *Masters or Professional degree (i.e. MBA, MPP, etc)*
   (g) *Doctoral Degree*
6. *What best describes your employment situation?*
   (a) *Full-time employed*
   (b) *Part-time employed*
   (c) *Unemployed*
   (d) *Caregiver (e.g., children, elderly) or homemaker*
   (e) *Retired*
   (f) *Full-time student*
   (g) *Other*
7. *What was your yearly personal income in 2018 (include salary, interests, returns on investments, etc)?*
   (a) *Less than $10,000*
   (b) *$10,000-$19,999*
   (c) *$20,000-$29,999*
   (d) *$30,000-$39,999*
   (e) *$40,000-$49,999*
   (f) *$50,000-$59,999*
   (g) *$60,000-$69,999*

(h) *$70,000-$79,999*
(i) *$80,000-$99,999*
(j) *$100,000-$119,999*
(k) *$120,000-$149,999*
(l) *$150,000-$199,999*
(m) *$200,000 - more*

8. *Which city do you live in?*
   - *Albuquerque, NM*
   - *Austin, TX*
   - *Baltimore, MD*
   - *Boston, MA*
   - *Charlotte, NC*
   - *Chicago, IL*
   - *Columbus, OH*
   - *Dallas, TX*
   - *Denver, CO*
   - *Detroit, MI*
   - *El Paso, TX*
   - *Fort Worth, TX*
   - *Fresno, CA*
   - *Houston, TX*
   - *Indianapolis, IN*
   - *Jacksonville, FL*
   - *Las Vegas, NV*
   - *Los Angeles, CA*
   - *Louisville, KY*
   - *Memphis, TN*
   - *Milwaukee, WI*
   - *Nashville, TN*
   - *New York City, NY*
   - *Oklahoma City, OK*
   - *Philadelphia, PA*
   - *Phoenix, AZ*
   - *Portland, OR*
   - *Sacramento, CA*
   - *San Antonio, TX*
   - *San Diego, CA*
   - *San Francisco, CA*
   - *San Jose, CA*
   - *Seattle, CA*
   - *Tucson, AZ*
   - *Washington, DC*
   - *Other:* [text box appears]
     (if "Other" selected, the participant is not eligible and is thanked and exits the survey)

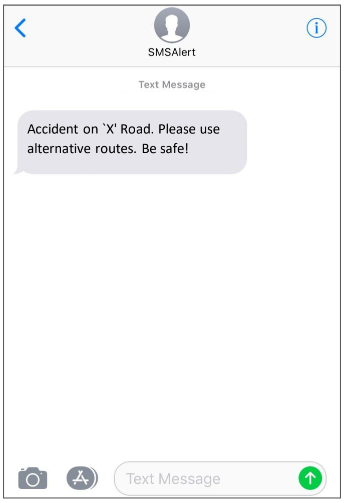

Figure S1: The traffic alert shown to the participants in our survey.

S1.1.3 Message with the fake notification

The following sentence was displayed to the participant:

- "*Suppose you receive the following message from SMSAlert*"

Below this text, the message shown in Fig. S1 was displayed to the participant.

S1.1.4 Questions to assess propensities to follow-through & forward notifications

1. *What is the likelihood that you use an alternative route?*

   [Likert scale with values: $0, 1, \ldots, 10$, where "0" is labeled at "never" and "10" is labeled as "definitely"]

2. *What is the likelihood that you forward this message to your friends?*

   [Likert scale with values: $0, 1, \ldots, 10$, where "0" is labeled at "never" and "10" is labeled as "definitely"]

S1.1.5 Closing message and compensation

This is the end of the study. Thank you for your participation. Please make note of the following 7-digit code. You will input it through Mechanical Turk to indicate your completion of the study. ***Then click the button on the bottom of this page to submit your answers. You will not receive credit unless you click this button.***

7-Digit Code: [code appears]

S1.2 Survey-2

We ran a second survey to understand how participants react to road signs and discount alerts. Here, we recruited 1,000 participants through Amazon Mechanical Turk, who were then directed to a survey on the Qualtrics platform. Each participant was a resident of one of the 35 cities that were considered in the previous survey from Section S1.1. The survey consisted of 8 steps, an overview of which is provided below. Detailed explanations of these steps are provided in Sections S1.2.1 through S1.2.7.

Step 1: A consent form is displayed, and is followed by a note to participants asking them to disregard any impact the COVID-19 pandemic may have on their responses to this survey (see Section S1.2.1). Subsequently, demographic questions are asked (see Section S1.2.2);

Step 2: The following sentence is displayed:

  – "*Suppose you are driving downtown, and you see this sign on the side of the road*";

Step 3: The message containing the traffic sign is displayed (see Section S1.2.3);

Step 4: The participant is asked questions regarding their follow-through propensity (see Section S1.2.4);

Step 5: The following sentence is displayed:

– "*Suppose you receive the following message from SMSAlert*";

Step 6: The message containing the fake discount alert is displayed (see Section S1.2.5);

Step 7: The participant is asked questions regarding their follow-through and forwarding propensities (see Section S1.2.6);

Step 8: The participant is awarded a financial compensation of $0.4, and thanked for their participation (Section S1.2.7).

In this survey, we randomized the order in which the two scenarios were presented to the participants. That is, some participants were first asked about the road sign (steps 2—4) and subsequently the discount alert (steps 5—7), while others were first asked about the discount alert (steps 5—7) followed by the road sign (steps 2—4).

S1.2.1 Consent form

The consent form consists of the following text:

Welcome to this study investigating how humans react to notifications. You are eligible to participate in the study at this time if you are:

1. *18 years of age or older;*
2. *live in one of the following 35 cities: Albuquerque (NM), Austin (TX), Baltimore (MD), Boston (MA), Charlotte (NC), Chicago (IL), Columbus (OH), Dallas (TX), Denver (CO), Detroit (MI), El Paso (TX), Fort Worth (TX), Fresno (CA), Houston (TX), Indianapolis (IN), Jacksonville (FL), Las Vegas (NV), Los Angeles (CA), Louisville (KY), Memphis (TN), Milwaukee (WI), Nashville (TN), New York City (NY), Oklahoma City (OK), Philadelphia (PA), Phoenix (AZ), Portland (OR), Sacramento (CA), San Antonio (TX), San Diego (CA), San Francisco (CA), San Jose (CA), Seattle (WA), Tucson (AZ), or Washington (DC).*

The questionnaire asks about your background and your reaction upon receiving notifications. This survey is anonymous, i.e., it does not contain individually identifiable data from you. Your participation is voluntary, and you may close the survey at any point.

The questionnaire is expected to last on average 1.5 minutes. An amount of $0.4 will be paid upon successful completion of the survey.

This research is conducted by Talal Rahwan, Bedoor AlShebli, and Marcin Waniek at New York University Abu Dhabi (NYUAD), and has been approved by NYUAD's Institutional Review Board. For questions about the rights of research participants, you may contact the University Committee on Activities Involving Human Subjects, New York University Abu Dhabi, irbnyuad@nyu.edu.

If you have any questions, suggestions or concerns, please feel free to reach out to us at nyuad.textnotification@nyu.edu – an email address that only researchers associated with this project have access to.

Please do not complete the survey more than once. Upon finishing the survey you will receive a completion code. The payment of $0.4 will be made once you've entered that code in the space provided. ***Please do not close the browser with your MTurk account****. If you read this consent form, and would like to participate in this study, press the button below!*

In the next page, the following note is displayed:

IMPORTANT: You will be asked questions about driving and traffic. Please describe how you would react in normal circumstances, without paying attention to any restrictions imposed due to the Coronavirus (COVID-19). In other words, please completely disregard any potential lockdown or travel restrictions when providing your answers.

S1.2.2 Demographic questions

The demographic questions that were asked to each participant are detailed below:

1. *What is your age?*

   [empty field to be filled by a number]

2. *Which city do you live in?*

   - *Albuquerque, NM*
   - *Austin, TX*
   - *Baltimore, MD*
   - *Boston, MA*
   - *Charlotte, NC*
   - *Chicago, IL*
   - *Columbus, OH*
   - *Dallas, TX*
   - *Denver, CO*
   - *Detroit, MI*
   - *El Paso, TX*
   - *Fort Worth, TX*
   - *Fresno, CA*
   - *Houston, TX*
   - *Indianapolis, IN*
   - *Jacksonville, FL*
   - *Las Vegas, NV*
   - *Los Angeles, CA*
   - *Louisville, KY*
   - *Memphis, TN*
   - *Milwaukee, WI*
   - *Nashville, TN*
   - *New York City, NY*
   - *Oklahoma City, OK*
   - *Philadelphia, PA*
   - *Phoenix, AZ*
   - *Portland, OR*
   - *Sacramento, CA*
   - *San Antonio, TX*
   - *San Diego, CA*
   - *San Francisco, CA*
   - *San Jose, CA*
   - *Seattle, CA*
   - *Tucson, AZ*
   - *Washington, DC*
   - *Other:* [text box appears]
     (if "Other" selected, the participant is not eligible and is thanked and exits the survey)

S1.2.3 Message with the traffic sign

The following sentence was displayed to the participant:

- "*Suppose you are driving downtown, and you see this sign on the side of the road:*"

Below this text, the message shown in Fig. S2 was displayed to the participant.

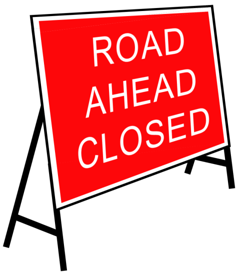

Figure S2: The traffic sign shown to the participants in our survey.

S1.2.4 Questions to assess propensity to follow-through

1. *What is the likelihood that you use an alternative route?*

   [Likert scale with values: $0, 1, \ldots, 10$, where "0" is labeled at "never" and "10" is labeled as "definitely"]

S1.2.5 Message with the fake discount

The following sentence was displayed to the participant:

- "*Suppose you receive the following message from SMSAlert:*"

Below this text, the message shown in Fig. S3 was displayed to the participant.

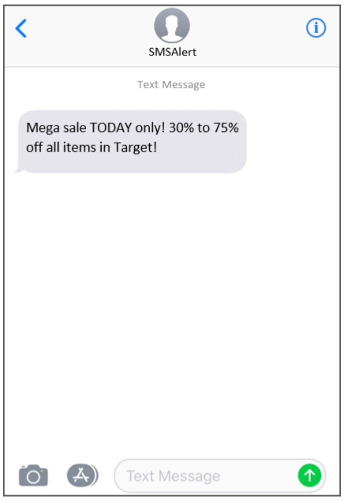

Figure S3: The discount notification shown to the participants in our survey.

S1.2.6 Questions to assess propensities to follow-through & forward notifications

1. *What is the likelihood that you drive to Target to take advantage of this offer?*

   [Likert scale with values: $0, 1, \ldots, 10$, where "0" is labeled at "never" and "10" is labeled as "definitely"]

2. *What is the likelihood that you forward this message to your friends?*

   [Likert scale with values: $0, 1, \ldots, 10$, where "0" is labeled at "never" and "10" is labeled as "definitely"]

S1.2.7 Closing message and compensation

This is the end of the study. Thank you for your participation. Please make note of the following 7-digit code. You will input it through Mechanical Turk to indicate your completion of the study. ***Then click the button on the bottom of this page to submit your answers. You will not receive credit unless you click this button.***

7-Digit Code: [code appears]

S2 Note 2: Road network and ride generation

This section details how the road network and vehicle rides in Chicago were generated.

S2.1 Road network generation

We obtained the road network data of Chicago from OpenStreetMap (OSM) [1]. However, we could not directly utilize this data for our simulations since: (i) some parts of the network were either disconnected or weakly connected due to the fact that OSM is crowdsourced and some streets were left unreported; (ii) the data did not contain information about the number of lanes in each edge of the network, which is required in our traffic model; and (iii) a section of a road may be represented by multiple edges in OSM instead of a single edge, making the corresponding graph needlessly large, thereby increasing the processing time in our simulations. Based on these observations, we developed an algorithm that takes the OSM data as input and generates a road network that addresses all of the aforementioned issues. The steps of the algorithm are as follows:

1. Create the set of nodes of the road network by extracting from OSM all nodes belonging to ways within a given area such that the value of the way's *highway* key is one of the following: *motorway*, *trunk*, *primary*, *secondary*, *tertiary*, *unclassified*, *residential*, or *service*. For each pair of nodes connected with a way in the OSM data, connect them in the road network with a single directed edge if the way's set of tags contains the key *oneway* with the value *yes*, *true* or *1*; otherwise, connect them with directed edges in both directions. For each such created edge, record its length defined as the geographical distance between the coordinates of the two nodes, computed using the haversine method [2]. Moreover, set the number of lanes of the edge based on the value of the way's *highway* key as follows:
   - For *motorway* or *trunk*, set the number of lanes to 4;
   - For *primary* or *secondary*, set the number of lanes to 3;
   - For *tertiary* or *unclassified*, set the number of lanes to 2;
   - For *residential* or *service*, set the number of lanes to 1.
2. Merge nearby nodes—defined here as being within 20 meters of each other—into a single node. Let $G' = (V', E')$ be the network before merging any nodes, where V' is the set of nodes and E' is the set of edges, and let $G = (V, E)$ be the network after merging the nodes. For each node $x \in V$ created by merging a group of nodes $X \subset V'$, set its coordinates to the average of the coordinates of all the nodes in X. Now, suppose we merged a group of nodes $X \subset V'$ into a single node $x \in V$, and merged another group $Y \subset V'$ into a node $y \in V$. Then, we add an edge $(x, y) \in E$ if and only if $\exists v \in X, u \in Y : (v, u) \in E'$, in which case we set the length of the edge to the geographical distance between the coordinates of x and y, and set the number of lanes of the edge to the maximum out of all the edges in $X \times Y$.
3. Ensure strong connectivity of the network by repeating the following process:
   (a) Select two strongly connected components $G_1 = (V_1, E_1)$ and $G_2 = (V_2, E_2)$ from the road network such that the geographical distance between the closest pair of nodes $x \in V_1$ and $y \in V_2$ is minimal;
   (b) Out of the edges (x, y) and (y, x), add the missing one(s) to E. Note that at least one of them is missing from E, because otherwise G_1 and G_2 would be a single strongly connected component.
   (c) Set the length of both (x, y) and (y, x) to the geographical distance between x and y;
   (d) If one of the edges (x, y) or (y, x) existed before step (b), set the number of lanes of the other edge to the number of lanes of the existing edge; otherwise set the number of lanes of both (x, y) and (y, x) to 1.

   This process is repeated until the road network becomes strongly connected. Note that this step is necessary since the OSM road network is often disconnected or weakly connected (due to the fact that OSM is crowdsourced and some roads may be left unreported), whereas the real road network of a city is strongly connected.
4. To make the computation more efficient, contract the edges of the road network, i.e., iteratively remove every node y that satisfies one of the following conditions:
   - Has exactly one predecessor x and exactly one successor z, such that $x \neq z$;
   - Has exactly two predecessors, which are also its only two successors.

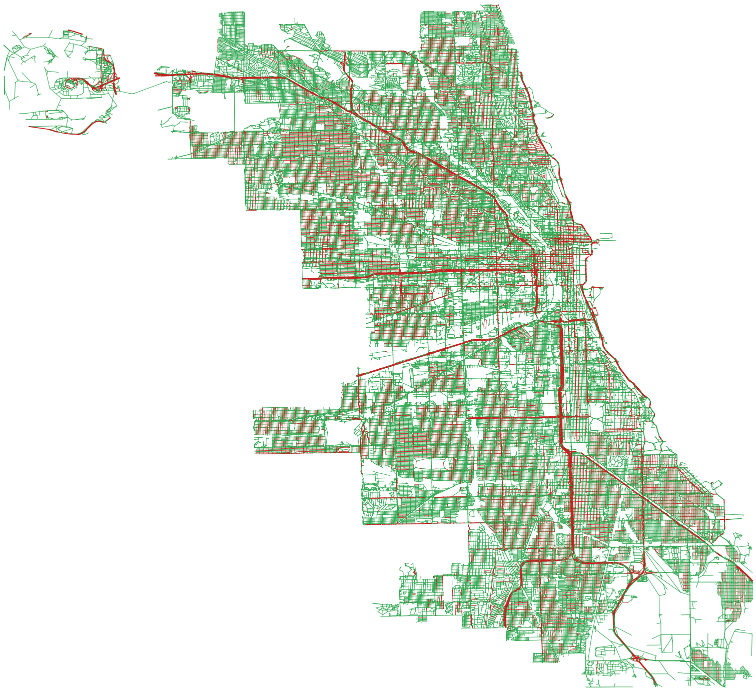

Figure S4: The road network of Chicago generated by our algorithm based on OSM data. Green edges represent two-way streets, while red edges represent one-way streets.

For example, if node y is connected only to nodes x and z, then replace the edges (x, y) and (y, z) with the edge (x, z). The length of the new edge is set to be the sum of lengths of (x, y) and (y, z), while the number of lanes of the edge is set to be the maximum of the numbers of lanes of (x, y) and (y, z).

Fig. S4 presents the road network of Chicago that was generated using the above algorithm while considering the OSM relation #122604 as the area boundaries.

S2.2 Ride generation

We now describe the algorithm that we developed to generate vehicle rides for our traffic simulations. The input to the algorithm is the following:

- The directed road network $G = (V, E)$ generated using the algorithm described in Section S2.1, where each edge has an assigned length and number of lanes;
- Data about the city traffic in the form of a set of pairs $D = \{(c_1, k_1), \dots, (c_{|D|}, k_{|D|})\}$, where c_i is a location (represented by its geographical coordinates), and k_i is the daily average of the number of vehicles passing by the location c_i;
- Distribution Θ of the intensity of the traffic throughout the day, measured by the number of rides that have started but have not yet reached their destination.

For the purpose of simulating the traffic in Chicago, G is generated taking the OSM relation #122604 as the area boundaries, D is generated using the data for October (the month with the greatest number of data points) provided by the city of Chicago [3], while Θ is based on the data provided by the Texas A&M Transportation Institute for Chicago [4] (we use the average taken over all weekdays).

The output of the algorithm is the set of rides R, where each ride is characterized by:

- Start node, $w^{start} \in V$;
- End node, $w^{end} \in V$;
- Time of day when the journey starts, θ.

Algorithm 1 presents the high-level pseudocode for generating the rides. The detailed steps are as follows:

Algorithm 1 Ride generation.

Input: Road network $G = (V, E)$; traffic data $D = \{(c_1, k_1), \ldots, (c_{|D|}, k_{|D|})\}$; traffic intensity distribution Θ
Output: Set of rides R

```
X ← {v_i ∈ V : D(v_i) ≠ ∅}        ▷ Identify the set of nodes in G that are closest to at least one set of coordinates c_j
for v_i ∈ X do
    x_i ← (Σ_{(c_j,k_j)∈D(v_i)} k_j) / |D(v_i)|        ▷ Initialize x_i to the average k_j in D(v_i)
Π ← ∅        ▷ Initialize the set of shortest paths from which the rides will be sampled
for v, w ∈ V do
    Let π be a shortest path from v to w in G
    if π ∩ X ≠ ∅ then        ▷ If π contains at least one sensor
        With probability p_load add π to Π
R ← ∅        ▷ Initialize the set of generated rides
while ∃_{v_i∈V} x_i > 0 ∧ Π ≠ ∅ do        ▷ Repeat until the generated rides match the sensor data or we run out of paths
    Draw π* from Π according to the distribution N(μ, σ)
    for v_i ∈ π* ∩ X do        ▷ Repeat for every sensor that appears on π*
        x_i ← x_i − 1        ▷ The number of rides that are supposed to pass by v_i is decreased by 1
        if x_i = 0 then        ▷ If the generated rides match the sensor data
            Π ← Π \ {π ∈ Π : v_i ∈ π}        ▷ Remove from Π all the shortest paths that contain v_i
    Draw θ from Θ        ▷ Draw the start time of the ride according to the daily traffic intensity distribution
    R ← R ∪ {(π*_1, π*_{|π*|}, θ)}        ▷ Add the generated ride (π*_1, π*_{|π*|}, θ) to the set of rides R
return R
```

1. **Initialize the number of vehicles passing through different nodes:** Let $D(v_i) \subseteq D$ be the set of pairs (c_j, k_j) for which $v_i \in V$ is the geographically closest node to the coordinates c_j. More formally, $D(v_i) = \{(c_j, k_j) \in D : v_i = \arg\min_{w \in V} \mathit{distance}(w, c_j)\}$. Moreover, let $X \subseteq V$ be the set of nodes that are closest to at least one set of coordinates. More formally, $X = \{v_i \in V : D(v_i) \neq \emptyset\}$. Note that for any given node $v_i \in X$, every pair $(c_j, k_j) \in D(v_i)$ indicates that the number of vehicles who passed by the location c_j is equal to k_j. Since this data was collected via a sensor, we will refer to the node v_i as a *sensor*. For every such sensor $v_i \in X$, let us denote by x_i the variable used in our simulation to count the number of rides who are supposed to go through v_i. This variable is initialized to the average value of k_j taken over all $(c_j, k_j) \in D(v_i)$. More formally,

$$x_i = \frac{\sum_{(c_j,k_j)\in D(v_i)} k_j}{|D(v_i)|}.$$

   Later on in our simulation, the value stored in any such variable x_i will be reduced by 1 every time a ride passes by the node v_i. Here, the goal is to reach a state at which the values x_i in all sensors are equal to zero, indicating that the number of vehicles who passed by v_i in our simulation matches the average number of vehicles who passed by the coordinates $c_j : (c_j, k_j) \in D(v_i)$.

2. **Select the subset of shortest paths Π:** For every pair of nodes $v, w \in V$ such that $v \neq w$, compute a shortest path π from v to w in G. If π contains at least one sensor, i.e., if $\pi \cap X \neq \emptyset$, then with probability $p_{load} = \frac{1}{1000}$ add π to the set Π. Paths in Π will be the only paths used to generate the set of rides R. In other words, instead of sampling the rides out of all possible shortest paths in the graph, we sample them out of those in Π to reduce the computational overhead and speed up the simulations. By sampling the rides from a subset of shortest paths, we ensure that every ride minimizes the travel distance (see Discussion in the main manuscript).

3. **Add a single path π^* to R and update the data structures:** Randomly select a shortest path $\pi^* = (\pi^*_1, \ldots, \pi^*_{|\pi^*|}) \in \Pi$. Here, every $\pi \in \Pi$ is selected with probability proportional to its length, according to a normal distribution $\mathcal{N}$ with mean $\mu = 8$ kilometers and standard deviation $\sigma = 1$ kilometer. For every sensor $v_i \in \pi^* \cap X$ decrease the value of x_i by 1. If for any $v_i \in \pi^* \cap X$ we now have $x_i = 0$, remove from Π every path π that goes through v_i. Add the ride $(\pi^*_1, \pi^*_{|\pi^*|}, \theta)$ to the set of rides R, where the starting time θ is selected according to the distribution Θ.

4. **Check the end condition:** Repeat step 3 until the generated rides match the sensor data or we run out of paths to be added, i.e., until $(\forall_{v_i \in V} x_i = 0) \vee (\Pi = \emptyset)$.

S3 Note 3: Our model of traffic M^*

Our model of traffic, M^*, is a modified version of the Nagel–Schreckenberg model [5]. We had to modify this model since it was only designed to model traffic in a single street, whereas our requirements call for modeling the traffic flows in a directed network of streets. We note that ours is not the first work to extend the Nagel–Schreckenberg model to a generic network. A similar extension was proposed by Gora [6] whose study focused on the role of traffic lights in managing the traffic flows. However, their traffic model was presented in rather vague terms, and therefore could not be used in our study.

We now describe our model in detail. The input to our traffic model M^* is the following:

- The directed road network $G = (V, E)$, where each edge $e \in E$ has an assigned length d_e and a number of lanes l_e;
- The set of rides $R = \{r_1, \ldots, r_{|R|}\}$, with each ride r_i being of the form $(w_i^{start}, w_i^{end}, \theta_i)$, where $w_i^{start} \in V$ is the start node, $w_i^{end} \in V$ is the end node, and θ_i is the time of day when the ride starts.

The model proceeds in discrete time steps. The time step corresponding to any given start time, θ_i, is denoted by $\tau(\theta_i)$. In other words, τ maps any given clock time to a particular time step in the model. For any edge, $e \in E$, the street associated with e is divided into a number of cells; each of which can be occupied by at most one vehicle in any given time step. The number of cells, c_e, in edge e is denoted by c_e and is computed as $c_e = \lceil \frac{d_e}{d_{vehicle}} \rceil$, where $d_{vehicle}$ is the average length of the space occupied by each vehicle on the road, including the separation between two consecutive vehicles on the same lane. Similar to the Nagel–Schreckenberg model, the speed of each ride, v_i, in our model is expressed as the number of cells it can traverse in a single time step. The maximum speed of all the rides is denoted by v_{max}.

Algorithm 2 presents a high-level pseudocode of the model. In words, for each time step t, the model involves the following actions:

1. For every ride, $(w_i^{start}, w_i^{end}, \theta_i) \in R$ whose starting time $\tau(\theta_i) = t$:
   - Add the ride to the set of ongoing rides Ω;
   - Compute the shortest path π_i from w_i^{start} to w_i^{end};
   - Set the state of the ride γ_i as waiting on the first edge of π_i;
   - Set the speed of the ride as $v_i = 0$.
2. For every ride $(w_i^{start}, w_i^{end}, \theta_i) \in \Omega$ that is waiting on some edge e, if there exists a lane λ of e with an empty first cell, put the ride in the first cell ($c_i = 1$) of λ, i.e., set $\lambda_i = \lambda$, and set the state of the ride as traversing edge e. In case there are multiple lanes with an empty first cell, then one of them is chosen uniformly at random to be λ_i.
3. For every ride $(w_i^{start}, w_i^{end}, \theta_i) \in \Omega$ that is traversing some edge e:
   (a) Increase the vehicle's speed by 1 if it is smaller than v_{max}, i.e., $v_i = \max(v_i + 1, v_{max})$.
   (b) If the current lane, λ_i, does not have at least v_i empty cells in front of the vehicle, change the lane to the one with the greatest number of empty cells, i.e.,
   $$\lambda_i = \underset{\lambda \in \{1,\ldots,l_e\}}{\arg\max}\ x_e(\lambda, c_i)$$
   where $x_e(\lambda, c_i)$ is the number of empty cells in front of cell c_i on lane λ of edge e.
   (c) Match the vehicle's speed to the number of empty cells in front of it in its current lane, i.e., $v_i = \min(v_i, x_e(\lambda_i, c_i))$.
   (d) If the vehicle's speed is greater than 0, then with a probability p_{slow} decrease it by 1, i.e., $v_i = \max(v_i - 1, 0)$.
   (e) Move the vehicle forward by v_i cells on its current lane, i.e., $c_i = c_i + v_i$. If the vehicle reached the last cell of e, remove it from e. If the vehicle got removed from e, either set its state to waiting at the next edge in π_i (if there are more edges in π_i), or remove it from the set of ongoing rides Ω (if it reached its final destination).

The process is continued until $t \geq t_{max}$ and all vehicles finish their journeys.

Algorithm 2 Model of traffic M^*.

Input: Road network $G = (V, E)$ where each edge $e \in E$ has an assigned length d_e and number of lanes l_e; a set of rides $R = \{(w_1^{start}, w_1^{end}, \theta_1), \ldots, (w_{|R|}^{start}, w_{|R|}^{end}, \theta_{|R|})\}$

$t \leftarrow 0$ ▷ Set the current time step to zero
$\Omega \leftarrow \emptyset$ ▷ Initialize the set of ongoing rides
while $t < t_{max} \vee \Omega \neq \emptyset$ **do**
  for $(w_i^{start}, w_i^{end}, \theta_i) \in R : \tau(\theta_i) = t$ **do** ▷ For every ride that starts at time t
    $\Omega \leftarrow \Omega \cup \{(w_i^{start}, w_i^{end}, \theta_i)\}$
    Let π_i be the shortest path from w_i^{start} to w_i^{end} in G
    $\gamma_i \leftarrow$ Waiting at $e_1(\pi_i)$ ▷ Set the status of the ride to "Waiting at the first edge of π_i"
    $v_i \leftarrow 0$ ▷ Set the speed of the ride to zero
  for $(w_i^{start}, w_i^{end}, \theta_i) \in \Omega : \gamma_i =$ Waiting at $e_j(\pi_i)$ **do** ▷ For every ride waiting at an edge in the network
    if there exists a lane λ in e with empty first cell **then**
      $\lambda_i \leftarrow \lambda$ ▷ Set the current lane of the ride to λ
      $c_i \leftarrow 1$ ▷ Set the ride's location to the first cell of λ_i
      $\gamma_i \leftarrow$ Traversing $e_j(\pi_i)$ ▷ Set the status of the ride to "Traversing the edge e_j"
  for $(w_i^{start}, w_i^{end}, \theta_i) \in \Omega : \gamma_i =$ Traversing $e_j(\pi_i)$ **do** ▷ For every ride traversing an edge in the network
    $e \leftarrow e_j(\pi_i)$ ▷ Let e denote the edge that the ride is traversing
    $v_i \leftarrow \max(v_i + 1, v_{max})$ ▷ Increase speed of the ride by 1 unless it has reached the maximum speed
    if $x_e(\lambda_i, c_i) < v_i$ **then** ▷ If there are less than v_i empty cells in front of the ride
      $\lambda_i \leftarrow \arg\max_{\lambda \in \{1,\ldots,l_e\}} x_e(\lambda, c_i)$ ▷ Reassign the ride to the lane with the most empty cells ahead
    $v_i \leftarrow \min(v_i, x_e(\lambda_i, c_i))$ ▷ Cap the speed to the number of empty cells
    $v_i \leftarrow \max(v_i - 1, 0)$ with probability p_{slow} ▷ With a probability of p_{slow}, slow down the ride by 1
    $c_i \leftarrow c_i + v_i$ ▷ Move the ride ahead by v_i cells
    if $c_i = c_e$ **then** ▷ If the ride has reached the last cell of the edge e
      if there exists an edge e^* after e in π_i **then** ▷ If the ride has not reached the end of π_i
        $\gamma_i \leftarrow$ Waiting at e^* ▷ Set the status of the ride to "Waiting at edge e^*"
      else
        $\Omega \leftarrow \Omega \setminus \{(\pi_i, \theta_i)\}$ ▷ Remove the ride from the set of ongoing rides
        $\gamma_i \leftarrow$ Finished ▷ Set the status of the ride to "Finished"
  $t \leftarrow t + 1$ ▷ Increase the time step by 1

S4 Note 4: Definitions

Definition S1 (The problem of Maximizing Disruption)**.** *The problem is defined by a tuple,* (G, Q, R, f, M, b)*, where* $G = (V, E)$ *is a directed network,* $Q \subseteq E$ *is the set of edges that the adversary can choose from,* R *is the set of rides,* f *is an objective function representing traffic quality,* M *is a model of traffic, and* $b \in \mathbb{N}$ *is the budget of the adversary. The goal is then to identify a set of targets that minimizes traffic quality, i.e., identify a set of edges* $Q^* \subseteq Q$ *in:*

$$\arg\min_{Q' \subseteq Q \;:\; |Q'| \leq b} f((V, E \setminus Q'), R, M).$$

Definition S2 (f^*)**.** *Given a graph* G*, a set of rides* R*, and a traffic model* M*, the objective function* f^* *is computed as:*

$$f^*(G, R, M) = \frac{1}{|R|} \sum_{r_i \in R} \frac{1}{\mathcal{T}(r_i, G, M)}$$

where $\mathcal{T}(r_i, G, M)$ *is the time taken to complete the ride* $r_i \in R$ *in the graph* G *according to the model* M*.*

Definition S3 ($M^{\varnothing}$)**.** *Given a graph* G*, and a set of rides* R *where every ride* $r_i \in R$ *travels from a starting node* $w_i^{start} \in V$ *to a destination node* $w_i^{end} \in V$*, the time of travel according to the simple traffic model* $M^{\varnothing}$ *is:*

$$\mathcal{T}(r_i, G, M^{\varnothing}) = d_G(w_i^{start}, w_i^{end})$$

where $d_G(w_i^{start}, w_i^{end})$ *is the number of edges on a shortest path between the two nodes, unless there exists no path between them, in which case* $d_G(w_i^{start}, w_i^{end}) = \infty$*.*

Definition S4 (The problem of Minimizing Targets)**.** *The problem is defined by a tuple,* (G, Q, R, f, M, ξ)*, where* $G = (V, E)$ *is a directed network,* $Q \subseteq E$ *is the set of edges that the adversary can choose from,* R *is the set of rides,* f *is an objective function representing traffic quality,* M *is a model of traffic, and* $\xi \in \mathbb{R}$ *is the attack efficiency. The goal is then to identify the smallest set of targets that decreases traffic quality below the threshold* ξ*, i.e., identify a set of edges* $Q^* \subseteq Q$ *in:*

$$\arg\min_{Q' \subseteq Q \;:\; f((V, E \setminus Q'), R, M) \leq \xi} |Q'|.$$

S5 Note 5: Theoretical results

In this section, we start by discussing works that are related to our theoretical analysis, before presenting our complexity results.

S5.1 Related works

From a theoretical perspective, the problem considered in our study involves minimizing the effectiveness of a certain process via the removal of some edges from a given network. A number of similar problems have been considered in the literature, usually related to the max-flow min-cut theorem [7]. These involve identifying the maximum amount of flow passing through the network, which can be interpreted as the amount of liquid traveling from the source in one node to the sink in another. In this context, the problem of minimizing the flow by removing edges from the network has been extensively studied [8], usually under the name of either *network inhibition* [9, 10] or *network interdiction* [11, 12]. Here, the network itself could be a representation of a logistic [13, 14], computer [15], or communication [16] system. This class of problems considers a process that is intrinsically continuous, whereas the cellular-automata model of traffic considered in our study is essentially discrete. For instance, a unit of liquid travelling from the source to the sink can be distributed across multiple network pipes, whereas a given vehicle in our traffic model cannot be broken into pieces that travel through different routes. Discrete variations of a similar problem were also considered, e.g., in the form of maximizing the distance between a given source-sink pair [17, 18], assuring that there does not exist a path between any two terminal nodes from a given set [19, 20] or disconnecting pairs of nodes [21, 22]. However, the problems considered thus far in the literature are typically defined in purely graph-theoretic terms and cannot readily be used in our context of traffic modeling, since they do not model essential aspects of traffic such as the externalities between different car rides and the temporal distribution of rides.

S5.2 Complexity analysis

Theorem 1. *The problem of Maximizing Disruption is NP-complete given the objective function f^* and the traffic model M^*.*

Proof. The decision version of the problem is as follows: given a directed road network $G = (V, E)$, the set of edges that the adversary can choose from $Q \subseteq E$, the set of rides R, the objective function f^*, the model of traffic M^*, the adversary's budget $b \in \mathbb{N}$, and the attack efficiency $\xi \in \mathbb{R}$, does there exist $Q^* \subseteq Q$ such that $|Q^*| \leq b$, and

$$f^*((V, E \setminus Q^*), R, M^*) \leq \xi.$$

The problem is trivially in NP, since computing the value of f^* after the removal of a given set of edges Q^* can be done in a polynomial time.

We will now prove that the problem is NP-hard. To this end, we will show a reduction from the NP-hard Minimum Multiway Cut problem. This problem is defined by a network (V, E) and a set of terminal nodes $S \subseteq V$. The goal is then to determine whether there exist c edges from E such that after removing these edges from G, there exists no path between any two terminal nodes. This problem was proven to be NP-hard for directed unweighted graphs, given the number of terminal nodes $k \geq 2$ [19].

The main idea of our proof of NP-hardness is as follows. We will first construct an instance of the problem of Maximizing Disruption corresponding to the given instance of the Minimum Multiway Cut problem. We will then show that a solution to the constructed instance of the problem of Maximizing Disruption is also a solution to the given instance of the Minimum Multiway Cut problem. Hence, the existence of a polynomial time algorithm solving the problem of Maximizing Disruption would imply the existence of a polynomial time algorithm solving the NP-hard Minimum Multiway Cut problem, which is impossible unless P=NP.

Given an instance $((V, E), S, c)$ of the Minimum Multiway Cut problem, let us construct the following instance of the problem of Maximizing Disruption:

- $G = (V, E)$;
- $Q = E$, i.e., all edges can be chosen by the adversary;
- $R = \bigcup_{s,s' \in S : s' \neq s} \{(s, s', 0), (s', s, 0)\}$, i.e., for every pair of different terminal nodes $s, s' \in S$, we create a ride with the starting node $\alpha_i = s$ and the destination node $\beta_i = s'$, and another ride with the starting node $\alpha_i = s'$ and the destination node $\beta_i = s$, where all rides start at midnight, i.e., $\forall_i \theta_i = 0$;
- $f = f^*$;

- $M = M^*$;
- $b = c$;
- $\xi = 0$.

Moreover, let the parameters of our model of traffic M^* be the following:

- The length of every street is exactly the length of the vehicle, which results in every lane of every street having exactly one cell, i.e., $\forall_{e \in E} c_e = 1$;
- Every street has a number of lanes equal to the total number of rides, i.e., $\forall_{e \in E} l_e = |R|$;
- The maximum speed of each vehicle is 1, i.e., $v_{max} = 1$;
- The probability of slowing down is 0, i.e., $p_{slow} = 0$.

Let us now analyze the time it takes to complete each ride. Since the number of lanes of every street is equal to the total number of rides, no ride has to wait to enter the street, as there is always at least one empty lane (even if other lanes are occupied by all the remaining rides in R). The maximum speed of the vehicle is $v_{max} = 1$, and since the probability of randomly slowing down is $p_{slow} = 0$, every ride reaches its maximum speed in the first time step of the model (notice that for every ride the time of start is $\theta_i = 0$) and never changes its speed later on. Further, since every street has the length of just one cell, it always takes just one time step to traverse each street on the shortest path from the start to the destination. Therefore, for a given ride r_i, the time it takes to reach the destination is simply the distance from the starting node to the destination node expressed as the number of edges, i.e., $\mathcal{T}(r_i, G, M^*) = d_G(\alpha_i, \beta_i)$. Under these conditions, the objective function is:

$$f^*(G, R, M^*) = \frac{2}{|S|(|S|-1)} \sum_{s,s' \in S : s \neq s'} \left(\frac{1}{d_G(s, s')} + \frac{1}{d_G(s', s)} \right).$$

Now, we show that if there exists a solution Q^* to the given instance of the Minimum Multiway Cut problem, i.e., a set of c edges such that after the removal of Q^* there exists no path between any two terminal nodes, then it is also a solution to the constructed instance of the problem of Maximizing Disruption. Since all the starting and destination nodes in the problem of Maximizing Disruption are terminal nodes from the Minimum Multiway Cut problem, after the removal of Q^* there are no paths between them, and the distance between them is ∞. Therefore, after the removal of Q^*, we have

$$f^*((V, E \setminus Q^*), R, M^*) = \frac{2}{|S|(|S|-1)} \sum_{s,s' \in S : s \neq s'} \left(\frac{1}{\infty} + \frac{1}{\infty} \right) = 0.$$

Hence, Q^* is a solution to the constructed instance of the problem of Maximizing Disruption.

To complete the proof of the theorem, we now show that if there exists a solution Q^* to the constructed instance of the problem of Maximizing Disruption, then it is also a solution to the given instance of the Minimum Multiway Cut problem. Since Q^* is a solution, after the removal of Q^*, the value of f^* is zero. If for at least one pair of starting and destination nodes there would exist a path between them, the distance between them would be smaller than ∞, and the $\frac{1}{d_G(\alpha_i, \beta_i)}$ component for this pair would cause the value of f^* to be positive. Therefore, since Q^* is a solution to the constructed instance of the problem of Maximizing Disruption, there can be no pair of starting and destination nodes with a path between them in $(V, E \setminus Q^*)$. However, because of the way we constructed this instance, the pairs of starting and destination nodes are exactly all pairs of terminal nodes from the given instance of the Minimum Multiway Cut problem. Hence, there are no paths between the terminal nodes in $(V, E \setminus Q^*)$, and Q^* is a solution to the given instance of the Minimum Multiway Cut problem. This concludes the proof. □

Theorem 2. *The problem of Maximizing Disruption is NP-complete given the objective function f^* and the traffic model $M^{\varnothing}$.*

Proof. The decision version of the problem is as follows: given a directed road network $G = (V, E)$, the set of edges that the adversary can choose from $Q \subseteq E$, the set of rides R, the objective function f^*, the model of traffic $M^{\varnothing}$, the adversary's budget $b \in \mathbb{N}$, and the attack efficiency $\xi \in \mathbb{R}$, does there exist $Q^* \subseteq Q$ such that $|Q^*| \leq b$ and

$$f^*((V, E \setminus Q^*), R, M^{\varnothing}) \leq \xi.$$

The problem is trivially in NP, since computing the value of f^* after the removal of a given set of edges Q^* can be done in polynomial time.

We will now prove that the problem is NP-hard. To this end, we will show a reduction from the NP-hard Minimum Multiway Cut problem. This problem is defined by a network (V, E) and a set of terminal nodes $S \subseteq V$. The goal is then to determine whether there exist c edges from E such that after removing these edges from G, there exists no path between any two terminal nodes. This problem was proven to be NP-hard for directed unweighted graphs, given the number of terminal nodes $k \geq 2$ [19]. The main idea of the proof is the same as for the proof of Theorem 1.

Given an instance $((V, E), S, c)$ of the Minimum Multiway Cut problem, let us construct the following instance of the problem of Maximizing Disruption:

- $G = (V, E)$;
- $Q = E$, i.e., all edges can be chosen by the adversary;
- $R = \bigcup_{s,s' \in S : s' \neq s} \{(s, s'), (s', s)\}$, i.e., for every pair of different terminal nodes $s, s' \in S$ we create a ride with starting node $\alpha_i = s$ and destination node $\beta_i = s'$, and another ride with starting node $\alpha_i = s'$ and destination node $\beta_i = s$;
- $f = f^*$;
- $M = M^{\varnothing}$;
- $b = c$;
- $\xi = 0$.

The objective function is then:

$$f^*(G, R, M^{\varnothing}) = \frac{2}{|S|(|S| - 1)} \sum_{s,s' \in S : s \neq s'} \left(\frac{1}{d_G(s, s')} + \frac{1}{d_G(s', s)} \right).$$

Notice that this is the same form as the objective function in the proof of Theorem 1. Hence the reasoning follows the same as in the proof of Theorem 1. We repeat it here for the convenience of the reader.

First, we show that if there exists a solution Q^* to the given instance of the Minimum Multiway Cut problem, i.e., a set of c edges such that after the removal of Q^* there exists no path between any two terminal nodes, then it is also a solution to the constructed instance of the problem of Maximizing Disruption. Since all the starting and destination nodes in the problem of Maximizing Disruption are terminal nodes from the Minimum Multiway Cut problem, after the removal of Q^*, there are no paths between them, and the distance between them is ∞. Therefore, after the removal of Q^*, we have

$$f^*((V, E \setminus Q^*), R, M^{\varnothing}) = \frac{2}{|S|(|S| - 1)} \sum_{s,s' \in S : s \neq s'} \left(\frac{1}{\infty} + \frac{1}{\infty} \right) = 0.$$

Hence, Q^* is a solution to the constructed instance of the problem of Maximizing Disruption.

To complete the proof of the theorem, we now show that if there exists a solution Q^* to the constructed instance of the problem of Maximizing Disruption, then it is also a solution to the given instance of the Minimum Multiway Cut problem. Since Q^* is a solution, after the removal of Q^*, the value of f^* is zero. If for at least one pair of starting and destination nodes there would exist a path between them, the distance between them would be smaller than ∞, and the $\frac{1}{d_G(\alpha_i, \beta_i)}$ component for this pair would cause the value of f^* to be positive. Therefore, since Q^* is a solution to the constructed instance of the problem of Maximizing Disruption, there can be no pair of starting and destination node with a path between them in $(V, E \setminus Q^*)$. However, because of the way we constructed this instance, the pairs of starting and destination nodes are exactly all pairs of terminal nodes from the given instance of the Minimum Multiway Cut problem. Hence, there are no paths between the terminal nodes in $(V, E \setminus Q^*)$, and Q^* is a solution to the given instance of the Minimum Multiway Cut problem. This concludes the proof. □

Theorem 3. *The problem of Minimizing Targets is APX-hard given the objective function f^* and either traffic model M^* or $M^{\varnothing}$. In particular, the problem does not admit a polynomial-time approximation scheme (PTAS) unless P=NP.*

Proof. We will prove that the problem is APX-hard by showing an L-reduction from the Minimum Multi-Cut problem. This problem is defined by a network $G = (V, E)$ and a set of source-destination pairs of nodes $S = \{(s_1, t_1), \ldots, (s_{|S|}, t_{|S|})\} \subseteq V \times V$. The goal is to find a subset $F \subseteq E$ such that removing edges F from G disconnects every source s_i from its destination t_i, and the size of F is minimal. The Minimum Multi-Cut problem was shown to be APX-hard [21].

The L-reduction will have the form of two polynomial time computable functions p and q such that:

(a) p constructs an instance of the problem of Minimizing Targets based on an instance of the Minimum Multi-Cut problem;

(b) q constructs a solution to an instance of the Minimum Multi-Cut problem based on a solution of the problem of Minimizing Targets;

(c) For any instance of the Minimum Multi-Cut problem (G, S), an optimal solution Q^* to $p(G, S)$ is of the same size as an optimal solution F^* to (G, S), i.e., $|Q^*| = |F^*|$;

(d) For every solution Q' to $p(G, S)$, we have that $||F^*| - |q(Q')|| = ||Q^*| - |Q'||$, where F^* is an optimal solution to (G, S) and Q^* is an optimal solution to $p(G, S)$.

First, let us define the function p. Let (G, S) be an instance of the Minimum Multi-Cut problem. The constructed instance of the problem of Minimizing Targets is then $p(G, S) = (G, Q, R, f, M, \xi)$, where:

- The network G is the same as for the Minimum Multi-Cut problem instance;
- $Q = E$, i.e., all edges can be chosen by the adversary;
- $R = \bigcup_{(s_i,t_i)\in S}\{(s_i, t_i)\}$, i.e., for every pair of source-destination nodes $(s_i, t_i) \in S$, we create a ride with starting node $\alpha_i = s_i$ and destination node $\beta_i = t_i$;
- $f = f^*$;
- M is the model of traffic, which could be either the simple model $M^{\varnothing}$ or our model of traffic M^*;
- $\xi = 0$, i.e., the removed set of edges has to decrease the traffic efficiency to 0.

Moreover, in the case of our model of traffic M^*, let its parameters be as follows:

- The length of every street is exactly the length of the vehicle, which results in every lane of every street having exactly one cell, i.e., $\forall_{e\in E} c_e = 1$;
- Every street has the number of lanes equal to the total number of rides, i.e., $\forall_{e\in E} l_e = |R|$;
- The maximum speed of each vehicle is 1, i.e., $v_{max} = 1$;
- The probability of slowing down is 0, i.e., $p_{slow} = 0$.

The simple model of traffic $M^{\varnothing}$ does not need any additional parameterization.

Note that $p(G, S)$ is a correct definition of an instance of the problem of Minimizing Targets and it is computable in polynomial time (hence point (a) is fulfilled). Note also that under these assumptions, the value of the objective function is:

$$f^*(G, R, M) = \frac{1}{|S|} \sum_{(s_i,t_i)\in S} \frac{1}{d_G(s_i, t_i)}.$$

In case $M = M^{\varnothing}$, this formula follows directly from the definition of the model (see Definition S3). In case $M = M^*$, see the proof of Theorem 1 for an explanation of how the formula for the objective function follows from the set of model parameters chosen above.

Second, we define the function q as simply $q(Q') = Q'$, i.e., the set of edges forming a solution to the Minimum Multi-Cut problem is the same set of edges forming a given solution to the problem of Minimizing Targets.

Now, we will show that if Q' is a solution to $p(G, S)$, then $q(Q')$ is indeed a solution to the given instance (G, S) of the Minimum Multi-Cut problem (point (b)). Note that in order for Q' to be a solution to $p(G, S)$, there cannot be a single pair of source-destination nodes $(s_i, t_i) \in S$ such that there exists a path from s_i to t_i in $(V, E \setminus Q')$. If at least one such pair exists, then we have:

$$f^*((V, E \setminus Q'), R, M) \geq \frac{1}{|S| d_G(s_i, t_i)} > 0,$$

whereas a solution Q' has to decrease the traffic efficiency to zero (this is because $\xi = 0$). Hence, if there are no paths from the source to destination in $(V, E \setminus Q')$, then $q(Q') = Q'$ is also a solution to the given instance (G, S) of the Minimum Multi-Cut problem.

Now, we will show that for any instance of the Minimum Multi-Cut problem, (G, S), an optimal solution Q^* to $p(G, S)$ is of the same size as an optimal solution F^* to (G, S) (point (c)). We will show this by contradiction. Assume there exists an optimal solution Q^* to $p(G, S)$ smaller than the optimal solution F^* to (G, S). However, as we have shown in the paragraph above, the removal of Q^* from G disconnects all pairs in S, hence Q^* is also a solution to (G, S). As Q^* is smaller than F^*, F^* cannot be optimal. Similarly, assume there exists an optimal solution F^* to (G, S) smaller than an optimal solution Q^* to $p(G, S)$. However, as the removal of F^* from G disconnects all pairs in S, the value of the objective function is $f^*((V, E \setminus F^*), R, M) = 0$ and F^* is also a solution to $p(G, S)$. Since F^* is smaller than Q^*, it follows that Q^* cannot be an optimal solution to $p(G, S)$.

To complete the proof of the theorem, we will now show that for every solution Q' to $p(G, S)$ we have $||F^*| - |q(Q')|| = ||Q^*| - |Q'||$, where F^* is an optimal solution to (G, S) and Q^* is an optimal solution to $p(G, S)$ (point (d)). In the paragraph above, we showed that the optimal solutions to (G, S) and $p(G, S)$ are of the same size, i.e., $|F^*| = |Q^*|$. Moreover, notice that $q(Q') = Q'$. Hence, we have $||F^*| - |q(Q')|| = ||Q^*| - |Q'||$.

Therefore, we showed that functions p and q define an L-reduction from the Minimum Multi-Cut problem to the problem of Minimizing Targets, given the objective function f^* and either the simple model of traffic $M^{\varnothing}$ or our model of traffic M^*. Since the Minimum Multi-Cut problem is APX-hard [21], the problem of Minimizing Targets is also APX-hard, as L-reduction preserves APX-hardness.

Finally, it is well-known that if there exists a PTAS for some APX-hard problem, then P=NP [23]. Hence, the problem of Minimizing Targets does not admit a PTAS, unless P=NP. □

S6 Note 6: Larger versions of figures from the main manuscript

In this section, we present larger versions of Figs. 2(a), 2(b), and 2(c) from the main manuscript.

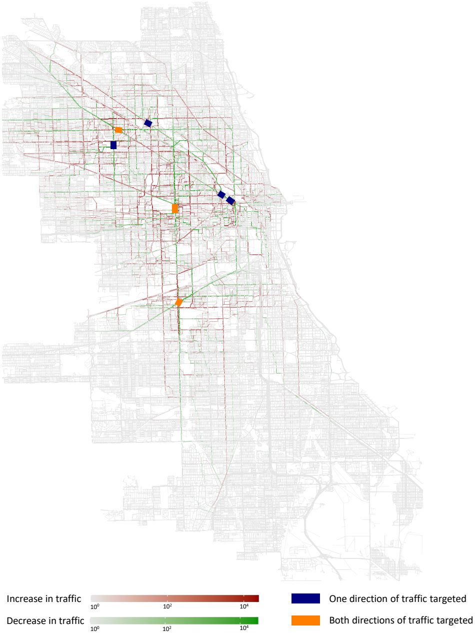

Figure S5: Larger reproduction of Fig. 2(a) in the main manuscript.

21

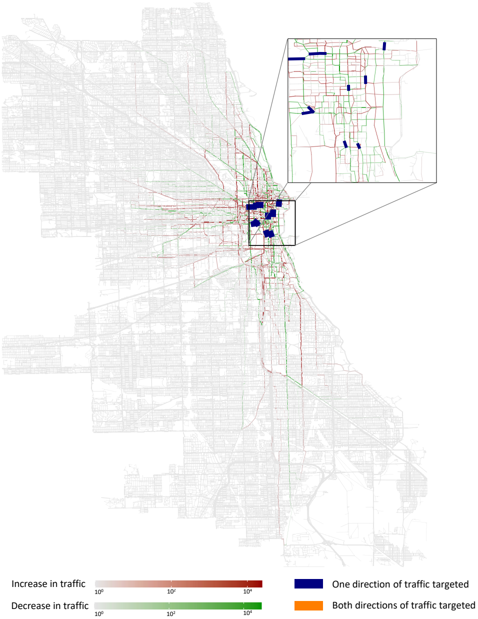

Figure S6: Larger reproduction of Fig. 2(b) in the main manuscript.

22

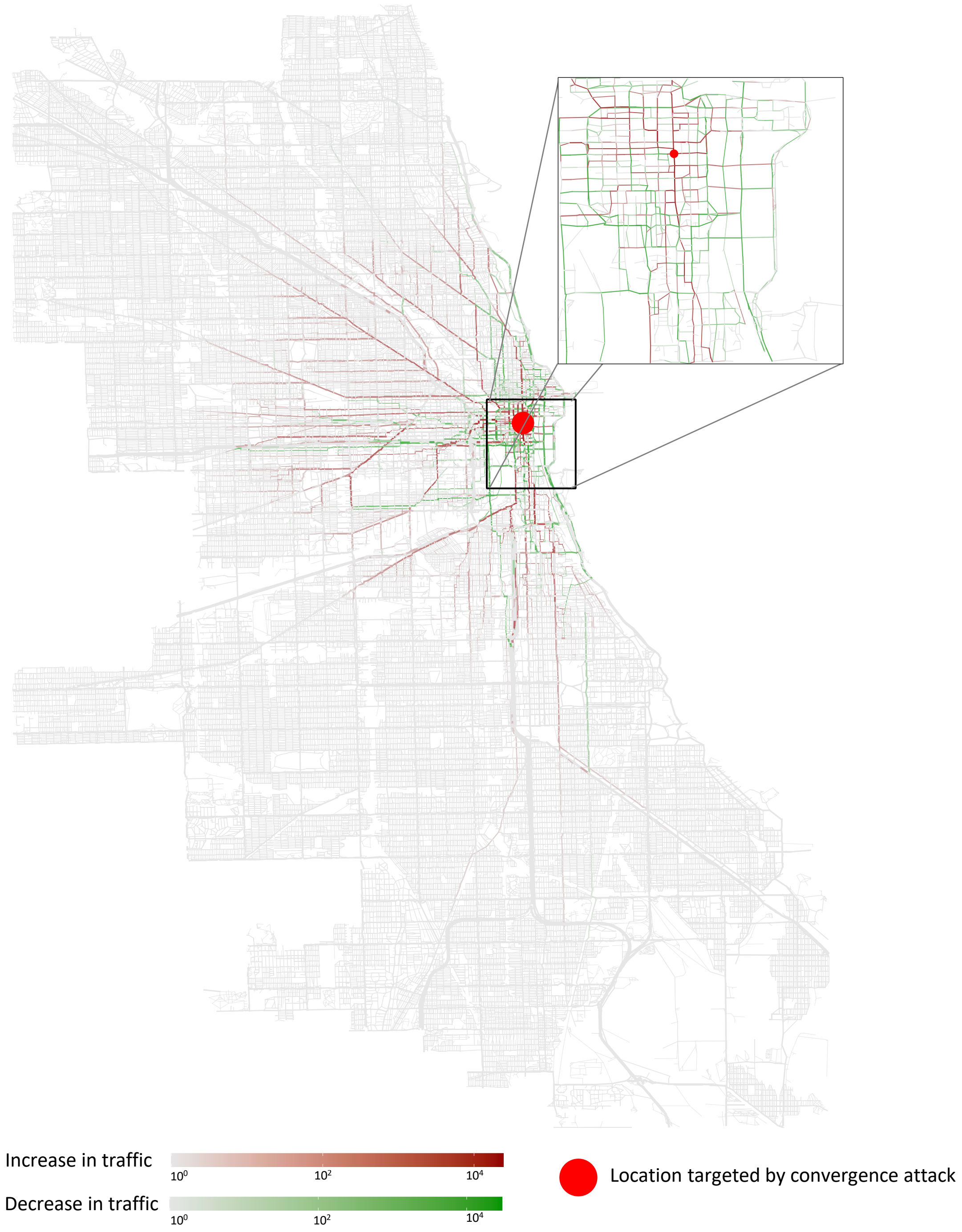

Figure S7: Larger reproduction of Fig. 2(c) in the main manuscript.

Supplementary References

[1] OpenStreetMap contributors. Planet dump retrieved from https://planet.osm.org. `https://www.openstreetmap.org`, 2019.

[2] Glen Van Brummelen. *Heavenly mathematics: The forgotten art of spherical trigonometry.* Princeton University Press, 2012.

[3] City of Chicago. Average daily traffic counts. `https://catalog.data.gov/dataset/average-daily-traffic-counts-3968f`, 2019. Accessed: 23.10.2019.

[4] Texas A&M Transportation Institute. Urban mobility report. `https://mobility.tamu.edu/umr/`, 2019. Accessed: 23.10.2019.

[5] Kai Nagel and Michael Schreckenberg. A cellular automaton model for freeway traffic. *Journal de physique I*, 2(12):2221–2229, 1992.

[6] Pawel Gora. Traffic simulation framework-a cellular automaton-based tool for simulating and investigating real city traffic. *Recent Advances in Intelligent Information Systems*, pages 641–653, 2009.

[7] Lester Randolph Ford and Delbert R Fulkerson. Maximal flow through a network. In *Classic papers in combinatorics*, pages 243–248. Springer, 2009.

[8] Richard Wollmer. Removing arcs from a network. *Operations Research*, 12(6):934–940, 1964.

[9] Cynthia A Phillips. The network inhibition problem. In *Proceedings of the twenty-fifth annual ACM symposium on Theory of computing*, pages 776–785, 1993.

[10] Carl Burch, Robert Carr, Sven Krumke, Madhav Marathe, Cynthia Phillips, and Eric Sundberg. A decomposition-based pseudoapproximation algorithm for network flow inhibition. In *Network Interdiction and Stochastic Integer Programming*, pages 51–68. Springer, 2003.

[11] R Kevin Wood. Deterministic network interdiction. *Mathematical and Computer Modelling*, 17(2):1–18, 1993.

[12] Kelly J Cormican, David P Morton, and R Kevin Wood. Stochastic network interdiction. *Operations Research*, 46(2):184–197, 1998.

[13] PM Ghare, Douglas C Montgomery, and WC Turner. Optimal interdiction policy for a flow network. *Naval Research Logistics Quarterly*, 18(1):37–45, 1971.

[14] Alan W McMasters and Thomas M Mustin. Optimal interdiction of a supply network. *Naval Research Logistics Quarterly*, 17(3):261–268, 1970.

[15] Tuomas Aura, Matt Bishop, and Dean Sniegowski. Analyzing single-server network inhibition. In *Proceedings 13th IEEE Computer Security Foundations Workshop. CSFW-13*, pages 108–117. IEEE, 2000.

[16] William H Cunningham. Optimal attack and reinforcement of a network. *Journal of the ACM (JACM)*, 32(3):549–561, 1985.

[17] Bruce Golden. A problem in network interdiction. *Naval Research Logistics Quarterly*, 25(4):711–713, 1978.

[18] Eitan Israeli and R Kevin Wood. Shortest-path network interdiction. *Networks: An International Journal*, 40(2):97–111, 2002.

[19] Naveen Garg, Vijay V Vazirani, and Mihalis Yannakakis. Multiway cuts in directed and node weighted graphs. In *International Colloquium on Automata, Languages, and Programming*, pages 487–498. Springer, 1994.

[20] David R Karger and Clifford Stein. A new approach to the minimum cut problem. *Journal of the ACM (JACM)*, 43(4):601–640, 1996.

[21] Elias Dahlhaus, David S. Johnson, Christos H. Papadimitriou, Paul D. Seymour, and Mihalis Yannakakis. The complexity of multiterminal cuts. *SIAM Journal on Computing*, 23(4):864–894, 1994.

[22] Marie-Christine Costa, Lucas Létocart, and Frédéric Roupin. Minimal multicut and maximal integer multiflow: a survey. *European Journal of Operational Research*, 162(1):55–69, 2005.

[23] Sanjeev Arora, Carsten Lund, Rajeev Motwani, Madhu Sudan, and Mario Szegedy. Proof verification and the hardness of approximation problems. *Journal of the ACM (JACM)*, 45(3):501–555, 1998.
